# Exploring the biomarkers for diagnostic accuracy associated with glycolysis and macrophage polarization in pediatric sepsis and performing mechanistic studies

**DOI:** 10.1097/MD.0000000000046074

**Published:** 2025-11-21

**Authors:** Hang Yu, Hui Sun, Jing Huang, Xiaoping Zhu

**Affiliations:** a Department of Paediatrics, Affiliated Hospital of Guizhou Medical University, Guiyang, Guizhou, China; b Medical Department, Guiyang Airport Emergency Center, Guiyang, Guizhou, China.

**Keywords:** biomarkers, glycolysis, immune infiltration, macrophage polarization, pediatric sepsis

## Abstract

The pediatric sepsis (PS) is characterized by severe clinical symptoms and high mortality. There was a lack of studies on the mechanisms of glycolysis and macrophage polarization (MP) in PS. The focus of this study was to identify biomarkers associated with glycolysis and MP in PS and to conduct mechanistic studies. The GSE26440 and GSE13904 datasets, glycolysis-related genes and macrophage polarization-related genes (MPRGs) were used in this study for analysis. First, to identify differentially expressed genes in GSE26440. Next, the weighted gene co-expression network analysis was taken to obtain key modular genes related between glycolysis and MP, and differentially expressed genes were overlapped with key modular genes to identify candidate genes. Subsequently, biomarkers were detected to biomarkers by constructing a protein–protein interaction network, machine learning and expression validation. Finally, based on the biomarkers, functional enrichment, regulatory network, immune microenvironment analysis, and the quantitative real-time polymerase chain reaction were analyzed. After screening, 4 biomarkers (a disintegrin and a metalloprotease9 [ADAM9], transforming growth factor alpha [TGFA], G protein subunit alpha q [GNAQ], and decaprenyl diphosphate synthase subunit 1 [PDSS1]) were obtained. The lysosome and fc gamma r mediated phagocytosis co-targeted 4 biomarkers in gene set enrichment analysis. Spearman correlation analysis showed that ADAM9, TGFA, GNAQ, and PDSS1 were significantly positively related to activated dendritic cells, and had a significantly negative correlation with activated B cells. Next, a long noncoding RNA (lncRNAs)-MicroRNAs (miRNAs)-mRNA network containing 7 microRNAs and 23 lncRNAs was constructed. The hsa-miR-302c-5P co-targeted ADAM9, TGFA, and PDSS1. Finally, quantitative real-time polymerase chain reaction showed that TGFA, GNAQ, and PDSS1 expression levels were significantly elevated in PS samples. In this study, 4 biomarkers (ADAM9, TGFA, GNAQ, and PDSS1) associated with glycolysis and MP were identified to provide scientific theories for the clinical management of PS.

## Graphical abstract

**Figure:**
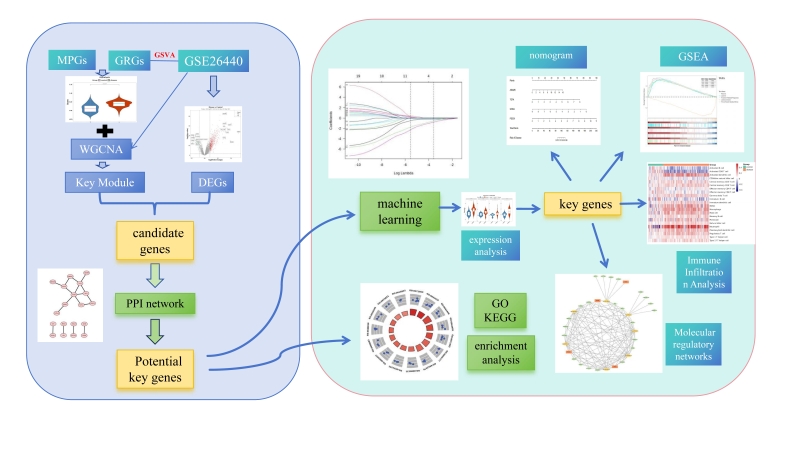

## 1. Introduction

In recent years, the global incidence and mortality of pediatric sepsis has been extremely high. More recently, it was reported that 1229 pediatric sepsis patients had a median age of 28 months, and only half of the children received a microbiologically confirmed diagnosis and had a high mortality rate.^[1]^ Currently, effective treatment is judged by decline in indicators of infection, reduction of symptoms, key principles of care include early identification of underlying sepsis, rapid intervention with appropriate fluid to restore adequate tissue perfusion, and empiric antibiotics may cover the pathogen.^[1–4]^ Despite this, the early assessment of the condition is still inadequate. Therefore, understanding the molecular mechanisms underlying PS is crucial for identifying early diagnostic biomarkers and the search for effective drugs.

Glycolysis is the central pathway of glucose metabolism, and its metabolite pyruvate can be converted into lactate, and it can also enter the mitochondria for tricarboxylic acid cycle and oxidative phosphorylation (OXPHOS). OXPHOS and glycolysis are the 2 major pathways for adenosine triphosphate production. The reliance on each varies across tissues and cell states, and can influence susceptibility to disease.^[5]^ In addition, glycolysis is the first choice for cells with high metabolic demand to meet the large energetic demands of metabolically active processes. Studies have shown that increased glycolysis in immune cells is closely associated with an excessive inflammatory response in sepsis. In the early stages of sepsis, the patient’s anti-infection response is activated and immune cells switch from mitochondria-dependent aerobic oxidation to a glycolysis-dominated anaerobic metabolism, thus responding to the rapid energy demand.^[6]^ Although glycolysis has been extensively studied in sepsis, the mechanisms underlying the genes associated with glycolysis in childhood sepsis are unclear.

Monocytes enter tissues and organs as macrophages and participate immune response of the body. Macrophages are natural immune cells that are highly plastic and have 2 extreme phenotypes of polarization: M1 and M2, and immunity is in balance when M1 and M2 are in equilibrium.^[7]^ In early sepsis, M1-like macrophage polarization (MP) releases large amounts of inflammatory factors, causing a severe inflammatory response. In contrast, in late sepsis, an increase in M2-like macrophages stimulates the release of anti-inflammatory cytokines and puts the host into a state of immunosuppression.^[8]^

Currently, there is a lack of studies on the mechanisms of glycolysis and MP genes in PS. Therefore, exploring glycolysis and MP genes will become a new target for the prevention and development of sepsis and prognosis, which is a new direction for the prevention and treatment of sepsis in children.

This study was based on bioinformatics and gene expression omnibus databases and identified biomarkers by differential analysis. Then, based on biomarkers, the functional enrichment, immune infiltration, regulatory network, and relative expression quantitative were analyzed. It is expected that this study will provide scientific evidence for the treatment of PS and give more active and effective diagnosis and treatment of the disease.

## 2. Materials and methods

Participants in this study were provided with a clear and understandable explanation of the research objectives, procedures, potential risks, and benefits. They were informed that their participation is voluntary and that they have the right to withdraw from the study at any time. Participants were given the opportunity to ask questions and provided written informed consent prior to their involvement in the study.

### 2.1. Study design and workflow

The overall workflow of this study is illustrated in Figure S1, Supplemental Digital Content, https://links.lww.com/MD/Q690. The dry lab component comprised differential expression analysis, WGCNA co-expression network construction, protein–protein interaction (PPI) network analysis, functional enrichment, machine learning-based biomarker screening, nomogram modeling, GSEA pathway analysis, and immune infiltration evaluation. The wet lab component involved clinical sample validation of candidate biomarkers through quantitative real-time polymerase chain reaction (qRT-PCR) to ensure research reliability.

### 2.2. Dry lab

#### 2.2.1. Data collection

The data associated with PS, GSE26440, and GSE13904 were sourced from the gene expression omnibus database (http://www.ncbi.nlm.nih.gov/geo/). In GSE26440 (GPL570), 98 PS (disease) and 32 control whole blood samples were included. In GSE13904 (GPL570), 52 PS and 18 control whole blood samples were included. Furthemore, 8 glycolysis-related genes (GRGs) were download from the molecular signatures database (https://www.gsea-msigdb.org/gsea/msigdb),^[9]^ and 35 macrophage polarization-related genes (MPRGs) were mined from the literature.^[10]^

#### 2.2.2. Differential expression analysis in GSE26440

To obtain the differentially expressed genes (DEGs) in the GSE26440 dataset, in silico differential expression analysis was taken by the “limma” package (version 3.54.0).^[11]^ The screening conditions were *P* < .05 and |log_2_ (FC)| > 1. Then, the DEGs were visualized by the “ggplot2” (version 3.4.4)^[12]^ and “pheatmap” packages (version 1.1.9)^[13]^ for volcano and heat maps, respectively.

#### 2.2.3. Weighted gene co-expression network analysis (WGCNA)

To screen the modular genes associated with glycolysis and macrophage polarization respectively, WGCNA was performed in this study using the “WGCNA” package (version 1.71).^[14]^ First, based on the PS and control samples in GSE26440 dataset, the “GSVA” package (version 1.46.0)^[15]^ was utilized to assess the GRGs-score and MPRGs-score respectively. Then, Wilcoxon test (*P* < .05) was utilized to analyze the differences in GRGs-score and MPRGs-score between the 2 samples. Next, all the samples in GSE26440 were hierarchically clustered to eliminate outlier samples. Subsequently, the optimal soft threshold (1–20) was searched. Set *R*^2^ = 0.85 to filter the soft thresholds that exceed the red cut line while the connectivity was close to 0. Later, a co-expression matrix was created to cluster the modules. The module merge parameter was 0.25. The minimum number of genes per module was 30. Additionally, the correlation of GRGs-score and MPRGs-score with each gene module was analyzed, and the results were plotted as heat maps. The key modules with significant correlation with both GRGs-score and MPRGs-score were identified (*|r*| > 0.3, *P* < .05), and the genes of the key modules were considered as key module genes.

#### 2.2.4. Protein–protein interaction (PPI) network

Using bioinformatic approaches, genes that crossed above DEGs and key module genes were noted as candidate genes. Then, to understand the interactions of the candidate genes between the corresponding proteins, a PPI network was created. The minimum value of the interaction score was at least 0.4 and the PPI network was visualized using “Cytoscape” (version 3.9.1).^[16]^ Next, the candidate genes with interactive relationships at the protein level were considered as candidate characteristic genes.

#### 2.2.5. Functional enrichment of candidate characteristic genes

Through computational functional annotation, the gene ontology (GO) and Kyoto Encyclopedia of Genes and Genomes enrichment analyses were taken in this study. Later, the significant pathways were plotted by the “enrichplot” package (version 1.10.2)^[17]^ and “GOplot” package (version 1.0.2),^[18]^ respectively. The screening conditions were all *P* < .05.

#### 2.2.6. Screening biomarkers

By applying machine learning algorithms, on the basis of candidate characteristic genes, the least absolute shrinkage and selection operator (LASSO) analysis was taken in this study. The characteristic genes were identified when the lambda value was taken as minimum. The support vector machine-recursive characteristic elimination (SVM-RFE) was proceed using the “caret” package (version 6.0-93).^[19]^ The genes corresponding to the points with the smallest error were used as the characteristic genes. Later, the characteristic genes derived from the 2 algorithms were crossed and recorded as key genes. Subsequently, the differences in expression of key genes in PS and control samples were compared by Wilcoxon test in GSE26440 and GSE13904. The results were plotted as box plots using the “ggplot2” package. The genes that were significantly differentially expressed (*P* < .05) and with the same expression trend between PS and controls in GSE26440 and GSE13904 were used as biomarkers.

#### 2.2.7. Construction and assessment of nomogram

Through statistical modeling, in all samples of the GSE26440 dataset, a nomogram was created via the “rms” R package (v 6.8-1; https://CRAN.R-project.org/package=rms). To appraise the performance of the nomogram, the “rms” (v 6.8-1) was utilized to produce the calibration curve. A calibration curve with a slope approaching 1 indicates a higher level of accuracy in the model’s predictive performance. Specifically, the Hosmer–Lemeshow (HL) test (*P* > .05) was the fitting index of the nomogram, which could be used to evaluate the discrepancy separating the predicted values from the actual values. Additionally, the “ggDCA” R package (v 1.1; https://CRAN.R-project.org/package=ggDCA) was applied to generate the decision curve analysis curves of the biomarkers and the nomogram. If they were above the “all” and “none” baseline lines, it indicated that the nomogram model had good clinical utility. Finally, the “pROC” R package (v 1.18.5; https://CRAN.R-project.org/package=pROC) was adopted to generate the ROC curve and calculate AUC value (AUC > 0.7).

#### 2.2.8. Gene set enrichment analysis (GSEA)

Employing pathway enrichment methodology, to clarify signaling pathways of biomarkers, spearman correlation scores were calculated between the biomarkers and other genes in the GSE26440 dataset using the “cor” function, and the genes were ranked (from high to low). Then, the sorted genes in the background gene set were subjected to GSEA using the “ClusterProfiler” package (version 4.7.1.003; *P* < .05).^[20]^ The background gene set was “c2.kegg.v7.4.symbols” and the “enrichplot” package was utilized to visualize the significantly enriched pathways (showing the Top5 enriched pathways).

#### 2.2.9. Functional network analysis

To further investigate functional associations of biomarkers, gene multiple association network integration algorithm (GeneMANIA; https://genemania.org/)^[21]^ was used to construct an integrated multi-omics interaction network of the biomarkers, incorporating various data types including PPIs, co-expression, pathway sharing, and genetic interactions. Significantly enriched pathways and biological functions were identified using a threshold of *P* < .05 (FDR < 0.05), with the interaction network visualized to reveal key biological relationships and functional modules.

#### 2.2.10. Immune infiltration analysis

Using deconvolution algorithms, infiltration of 28 immune cells in GSE26440 was assessed through the ssGSEA algorithm in the “GSVA” package. In the GSE26440 training set, the expression of 28 immune cells in the PS and control samples were compared by the wilcoxon test (*P* < .05). Subsequently, immune cells that were dramatically different between PS and control samples in the training set were screened (*P* < .05) and box plots were drawn. In addition, Spearman correlation analysis (|cor| > 0.3 and *P* < .05) was taken to analyze the relevance of the biomarkers and immune cells through the “psych” package (version 3.54.0).^[22]^ The correlation heat maps were drawn through the “corrplot” package (version 0.92).^[23]^

#### 2.2.11. Regulation network analysis

In this study, an integrated bioinformatics approach was employed using DNA intelligent analysis microT database (http://diana.imis.athena-innovation.gr/DianaTools/index.php?r=microT_CDS/index), miRanda database (https://www.mirbase.org/), and microcosm database (https://www.mirbase.org/) and microRNA DataBase database (http://www.mirdb.org) were used to predict microRNAs (miRNAs) targeting biomarkers. The intersection of miRNAs obtained from the 4 databases was taken as the differentially expressed miRNAs regulating the biomarkers. The long noncoding RNAs (lncRNAs) were predict in starbase database (http://starbase.sysu.edu.cn). Subsequently, the lncRNA-miRNA-mRNA network was constructed and visualized.

#### 2.2.12. Drug prediction and molecular docking

To delve into the interrelationships between biomarkers and potential drugs during the PS development, the drug-gene interaction database (https://dgidb.org/) was used to investigate the potential drugs associated with biomarkers, and a drugs–biomarkers interaction network was constructed by Cytoscape (v 3.10.2). In this study, drugs with the highest scores for their related effects on the biomarkers were selected for molecular docking, respectively. Subsequently, the uniprot website (https://www.uniprot.org/) was used to predict the 3D structures of the proteins linked to the biomarkers. The 3D structure files pertaining to the drugs were retrieved via the PubChem database (https://pubchem.ncbi.nlm.nih.gov/). Then, the 3D structures of the proteins and drugs were uploaded to the CB - Dock2 online website (https://cadd.labshare.cn/cb-dock/php/blinddock.php), and the binding free energy was calculated. In general, when |Total Score| was >5.0 kcal/mol, this demonstrated excellent binding ability between biomarkers and corresponding targeted drugs.

### 2.3. Wet lab

#### 2.3.1. The quantitative real-time polymerase chain reaction (qRT-PCR)

Experimental validation was conducted through qRT-PCR, the expression levels of biomarkers in GSE26440 were verified by qRT-PCR. A total of 20 blood samples (10 control and 10 PS) were acquired from the clinic in the Affiliated Hospital of Guizhou Medical University. All participants were given informed consent. The study had the approval of the Affiliated Hospital of Guizhou Medical University ethics committee (Additional file). First, total RNA was extracted from 20 samples using TRIzol reagent, followed by total RNA concentration and purity using a NanoPhotometer N50. The RNA was then reverse transcribed to synthesize cRNA, followed by 40 cycles of PCR. Finally, the relative expression of the genes was calculated using the 2^−ΔΔCt^ method. The internal reference was GAPDH, and the specific primer sequences were shown in Table S1, Supplemental Digital Content, https://links.lww.com/MD/Q690.

#### 2.3.2. Statistical analysis

The R package (version 4.2.2) was utilized for analysis of data. When *P* < .05, it meaned that the statistical significance was significant.

## 3. Results

### 3.1. Key research findings

The principal research findings of this study are summarized in Figure S2, Supplemental Digital Content, https://links.lww.com/MD/Q690.

### 3.2. The 43 candidate genes was screened

**Figure 1.**
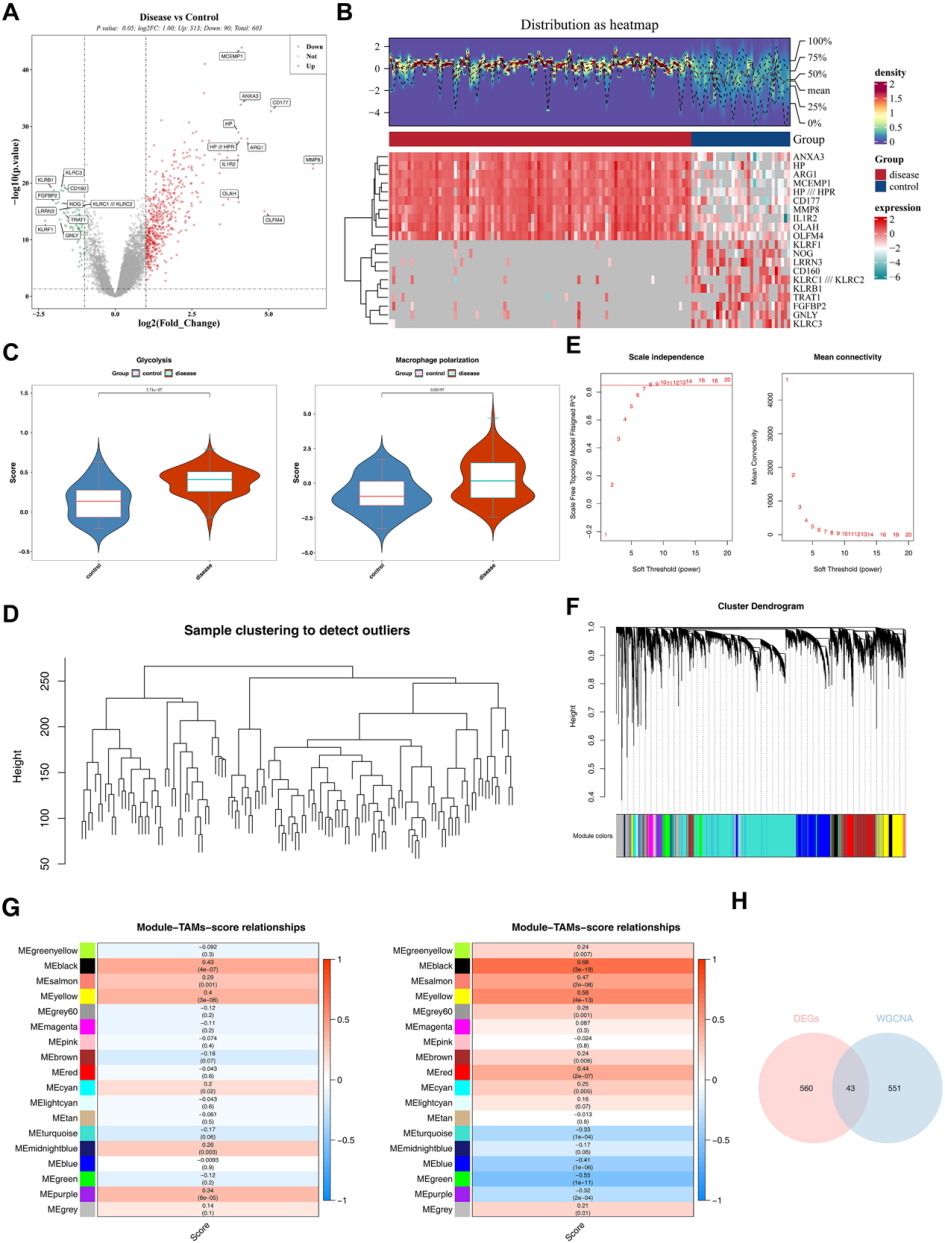
Screening candidate genes. (A, B) Volcano map of DEGs, and heatmap of the top 10 DEGs up- and down-regulated. (C) GRGs-score and MPRGs-score in GSE26440 (top: GRGs-score; bottom: MPRGs-score). (D) Clustering of all samples in the GSE26440 dataset. (E) Soft threshold power screening when *R*^2^ = 0.85. (F) The soft threshold power took the value of 9. A total of 18 co-expression modules were then identified. (G) Heatmap of correlation between modules and scores (top: GRGs-score and modules; bottom: MPRGs-score and module). (H) Intersection of DEGs with modular genes yields candidate genes. DEGs = differentially expressed genes, GRGs = glycolysis-related genes, MPRGs = MP-related genes.

The 603 DEGs were screened, of which 513 and 90 were up-regulated and down-regulated genes, respectively (Fig. 1A, B). Based on PS and control samples in GSE26440, by comparing the differences in scores for glycolysis and MP, GRGs-score and MPRGs-score were observed to be significantly different between 2 samples (*P* < .05), both of which were scored higher among PS samples (Fig. 1C). Hierarchical clustering of all samples revealed no outlier samples, so all samples were retained (Fig. 1D). As shown in Figure 1E, the soft threshold for filtering connectivity close to 0 was set at *R*_2_ of 0.85, and the soft threshold power took the value of 9. A total of 18 co-expression modules were then identified (Fig. 1F). In addition, by analyzing the association of GRGs-score and MPRGs-score with modular genes, the black module with the highest relevance was recorded as the key module, which included 594 key module genes (Fig. 1G). Finally, the above 603 DEGs and 594 key module genes were crossed and 43 candidate genes were identified (Fig. 1H).

### 3.3. Acquisition and functional enrichment analysis of candidate characteristic genes

**Figure 2.**
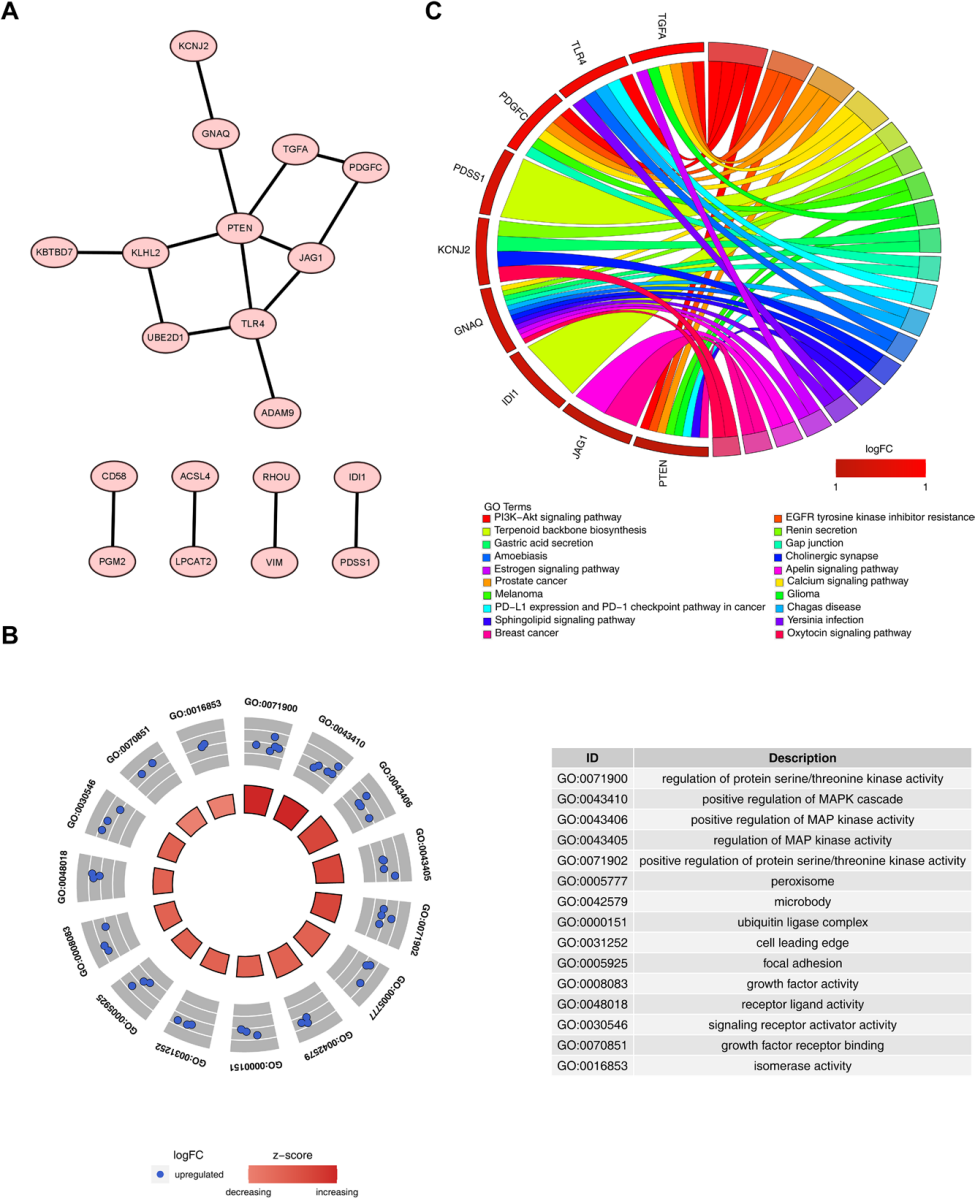
Acquisition of candidate characteristic genes and functional enrichment analysis. (A) The PPI network of candidate genes. (B) GO analysis of the 19 candidate characteristic genes. (C) 137 KEGG pathway enriched for candidate characteristic genes. GO = gene ontology, KEGG = Kyoto encyclopedia of genes and genomes, PPI = protein–protein interaction.

The PPI network included 19 nodes and 17 edges with interactions such as TLR4-PTEN and KLHL2-UBE2D1. The average node degree was 0.81, the local clustering coefficient was 0.276, and the enrichment *P*-value was .028. After performing gene screening, the corresponding proteins of the 19 candidate genes had interactions and were recorded as candidate characteristic genes (Fig. 2A). Then, a total of 514 entries were found to be enriched by GO analysis of the 19 candidate characteristic genes. In terms of biological processes, candidate characteristic genes were associated with regulation of positive regulation of mitogen-activated protein kinase (MAPK) cascade and positive regulation of MAP kinase activity. In cellular components, the candidate characteristic genes had a association with peroxisome, microbody, ubiquitin ligase complex, cell leading edge, and focal adhesion. In molecular functions, candidate characteristic genes were enriched to entries with growth factor activity, receptor ligand activity, signaling receptor activation activity, growth factor receptor binding, and isomerase activity (Fig. 2B). Furthermore, 137 pathways were enriched by candidate characteristic genes, including the PI3K-Akt signaling pathway, prostate cancer, terpenoid backbone biosynthesis, renin secretion, and melanoma, etc (Fig. 2C).

### 3.4. ADAM9, TGFA, GNAQ, and decaprenyl diphosphate synthase subunit 1 (PDSS1) were identified as biomarkers

**Figure 3.**
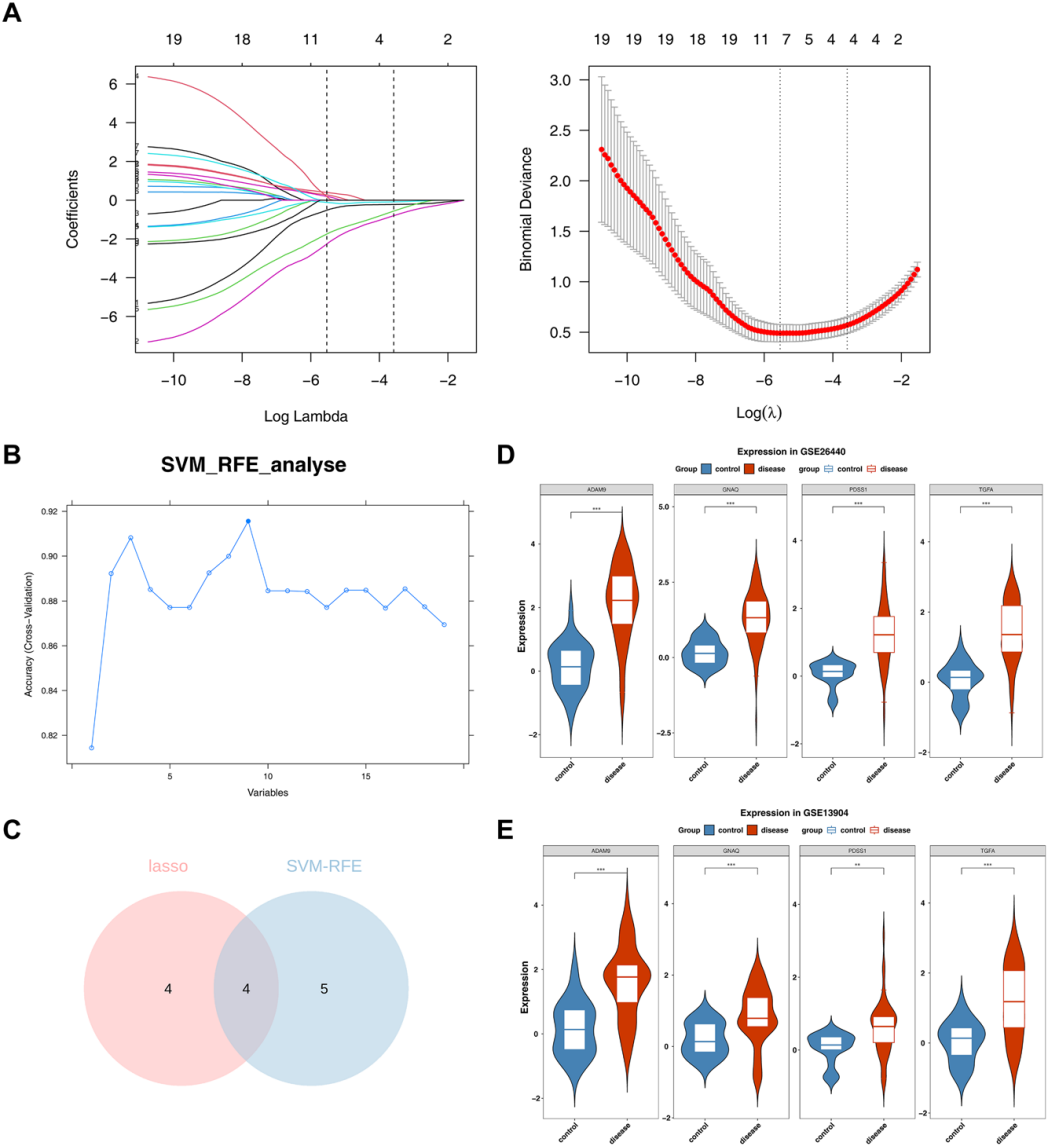
ADAM9, TGFA, GNAQ, and PDSS1 were identified as biomarkers. (A) Constructing a LASSO regression model for the candidate characteristic genes. (B) Identifying candidate characteristic genes by selecting points with the lowest error rate through SVM-RFE machine learning. (C) Intersection of LASSO and SVM-RFE as key genes. (D, E) Validation of expression of biomarkers in GSE26440 and GSE13904. ADAM9 = a disintegrin and a metalloprotease9, GNAQ = G protein subunit alpha q, LASSO = least absolute shrinkage and selection operator, PDSS1 = decaprenyl diphosphate synthase subunit 1, SVM-RFE = support vector machine-recursive characteristic elimination, TGFA = transforming growth factor alpha.

By constructing a LASSO regression model for the candidate characteristic genes, it was found that 8 characteristic genes (a disintegrin and a metalloprotease9 [ADAM9], TLR4, KBTBD7, UBE2D1, KCNJ2, TGFA, GNAQ, and PDSS1) were identified with a lambda value of 0.003947842 (at the lowest point of the curve; Fig. 3A). In SVM-RFE, 9 characteristic genes were identified by selecting the lowest point of error rate as the best combination, namely TGFA, ADAM9, GNAQ, PDSS1, CD58, PTEN, LPCAT2, VIM, and KLHL2 (Fig. 3B). Subsequently, the genes screened by the 2 algorithms were overlapped, and 4 genes, ADAM9, TGFA, GNAQ, and PDSS1, were finally identified to be recorded as key genes (Fig. 3C). Besides, the 4 key genes (ADAM9, TGFA, GNAQ, and PDSS1) were significantly differentially expressed and had consistent expression trends in both PS and control samples in GSE13904 and GSE26440, and the 4 key genes were recorded as biomarkers (Fig. 3D, E). Notably, ADAM9, TGFA, GNAQ, and PDSS1 were all highly expressed in PS samples.

### 3.5. The good nomogram was constructed and evaluated

**Figure 4.**
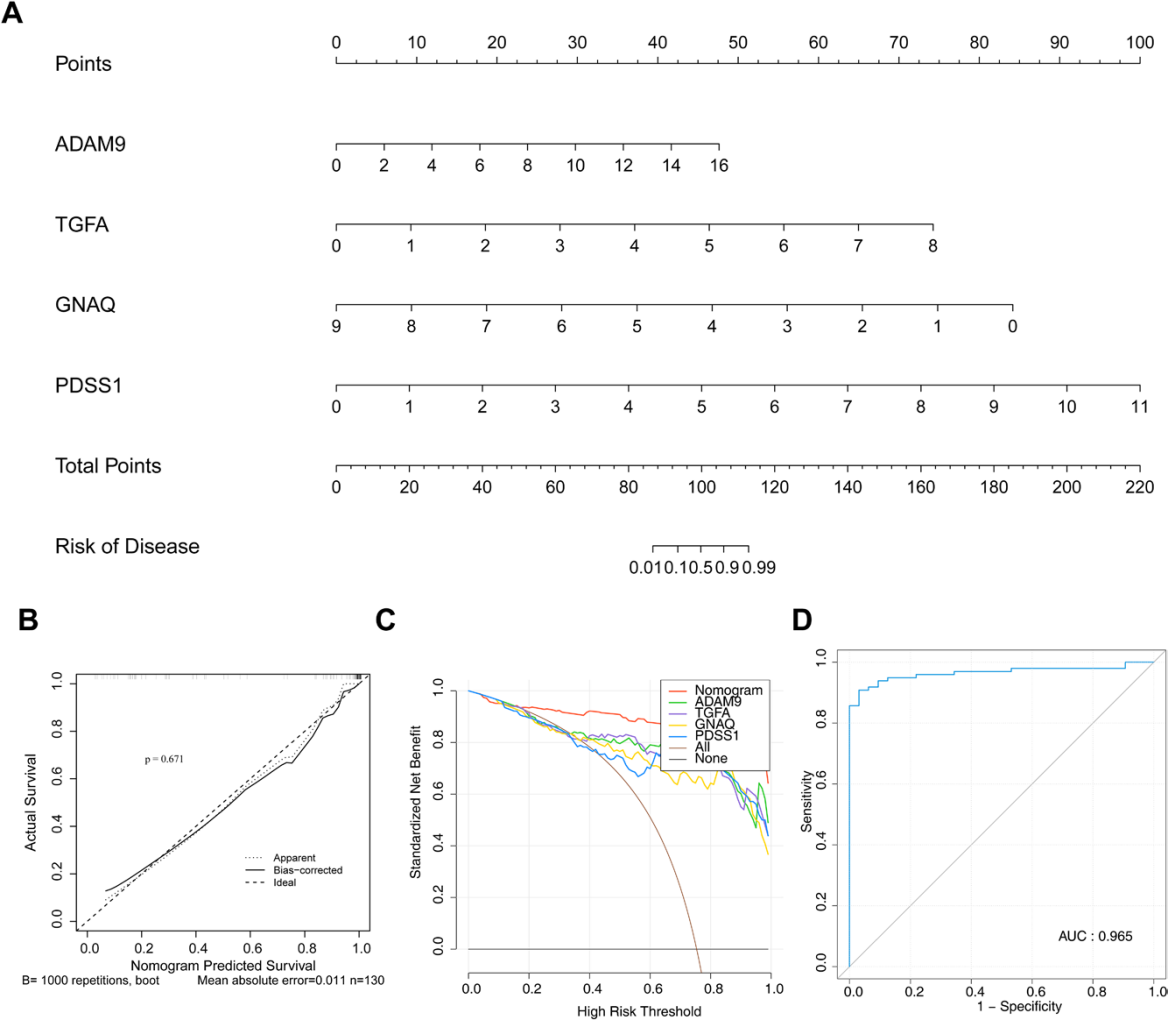
Construction of the nomogram. (A) The nomogram was constructed based on the biomarkers. (B) The calibration curve was used to validate the nomogram. (C) The decision curve analysis (DCA) curve was used to validate the nomogram. (D) The receiver operating characteristic (ROC) curve was used to validate the nomogram. DCA = decision curve analysis, ROC = receiver operating characteristic.

A good nomogram was constructed, which could clearly show the relationship between the total points of all biomarkers and the risk of PS development (Fig. 4A). Specifically, the *P* value of the HL test (*P* = .671) indicated that the prediction accuracy of the nomogram was relatively good (Fig. 4B). The results of the DCA curves showed that the curves of the biomarkers and the nomogram were above the “all” and “none” baseline lines, denoting that the nomogram exhibited good clinical utility (Fig. 4C). The receiver operating characteristic curve moreover suggested that the nomogram displayed a high predictive value (AUC = 0.965; Fig. 4D). The above results indicate that the nomogram has good accuracy, clinical utility, and high predictive value in predicting the risk of PS onset, providing an effective tool for the clinical risk assessment of PS.

### 3.6. Functional enrichment, functional network and immune infiltration analysis of biomarkers

**Figure 5.**
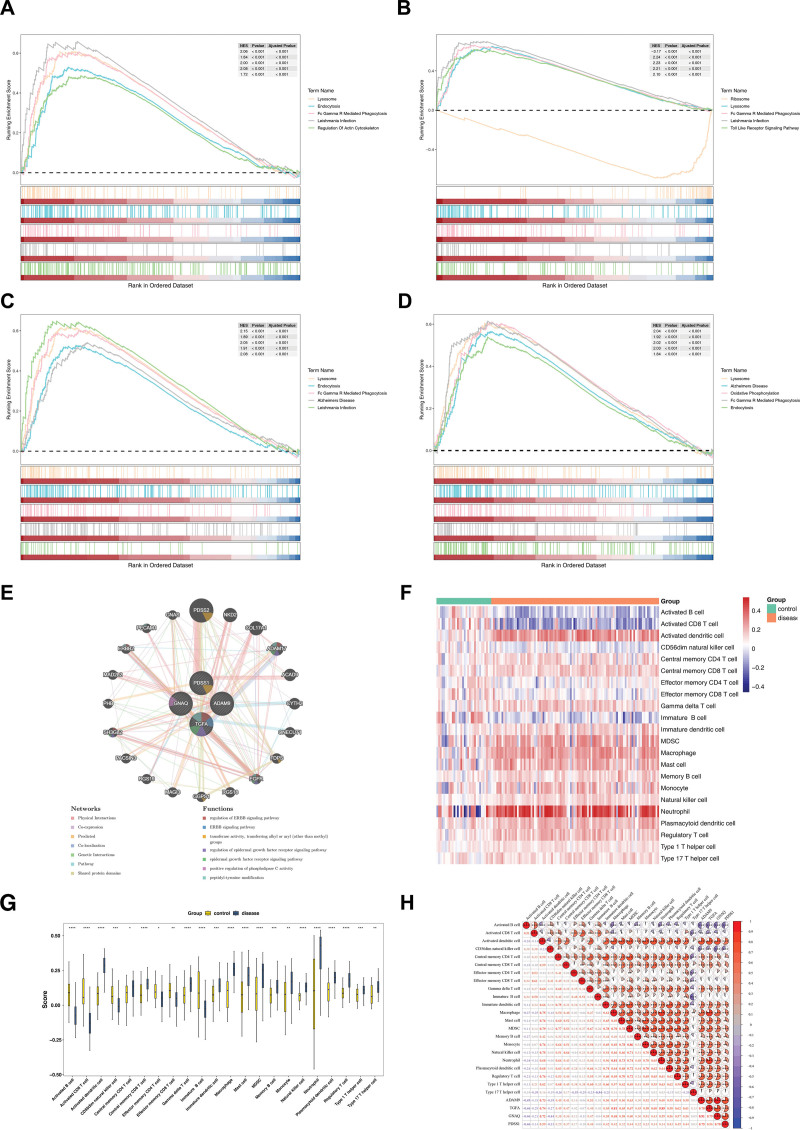
Functional and immune infiltration analysis of biomarkers. (A–D) GSEA-enriched pathways for biomarkers (ADAM9, TGFA, GNAQ, PDSS1). (E) In GSE26440, heat map showing infiltration of 28 immune cells. (F) Twenty-twenty two immune cells were significantly different in PS and control samples. (G) Heatmap of correlations between differential immune cells, between differential immune cells and biomarkers. GNAQ = G protein subunit alpha q, PDSS1 = decaprenyl diphosphate synthase subunit 1, TGFA = transforming growth factor alpha.

As shown in Figure 5A–D, the pathways enriched to by GNAQ were endocytosis, lysosome, and fc gamma r mediated phagocytosis. The ribosome, lysosome and fc gamma r mediated phagocytosis were the pathways enriched by TGFA. The ADAM9 was enriched to pathways such as lysosome, endocytosi and fc gamma r mediated phagocytosis. Lysosome, alzheimers disease and oxidative phosphorylation were pathways enriched for PDSS1. Notably, lysosome and fc gamma r mediated phagocytosis co-targeted 4 biomarkers.

Functional interaction analysis through GeneMANIA further expanded the protein network of biomarker (Fig. 5E). The biomarkers demonstrated multiple interaction relationships among themselves and with 20 functionally related genes, including physical interactions (77.64%), co-expression (8.01%), predicted functional associations (5.37%). Pathway enrichment analysis revealed significant involvement in ERBB signaling pathway (*P* < .05), regulation of the ERBB signaling pathway, epidermal growth factor receptor (EGFR) signaling pathway, and its regulation, along with positive regulation of phospholipase C activity. Functional annotation highlighted associations with transferase activity (transferring alkyl or aryl groups, other than methyl) and peptidyl-tyrosine modification process (FDR < 0.05).

In GSE26440, 28 immune cell correlations were shown in Figure 5F. Then, 22 immune cells were significantly different (*P* < .05) in PS and control samples. Among them, activated dendritic cells, MDSC and neutrophils had higher enrichment scores in PS samples than in control samples (Fig. 5G). Finally, Spearman correlation analysis showed that ADAM9, TGFA, GNAQ, and PDSS1 were significantly positively related to activated dendritic cells (*r* > 0.3, *P* < .05), and had a significantly negative correlation with activated B cells (*r* < −0.3, *P *< .05; Fig. 5H).

### 3.7. Regulatory network analysis was helpful for exploring molecular mechanism

**Figure 6.**
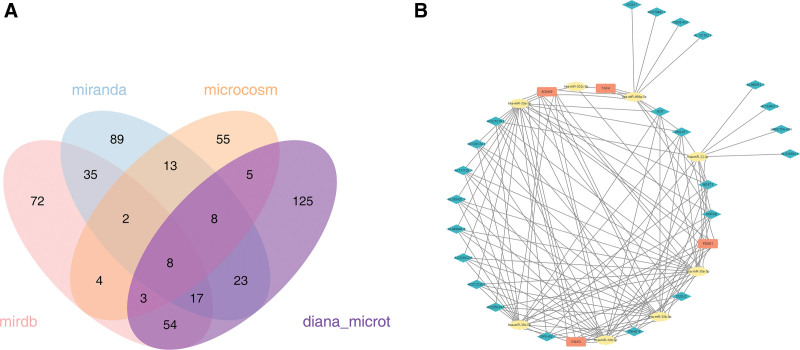
Prediction of miRNAs and lncRNAs and network construction. (A) Biomarker prediction of miRNA intersections. (B) lncRNA-miRNA-mRNA network. lncRNAs = long noncoding RNA, miRNAs = microRNAs.

A total of 8 miRNAs were obtained by predicting miRNAs for biomarkers. After that, lncRNAs were predicted and 7 miRNAs were found to predict 23 lncRNAs. Subsequently, the lncRNA-miRNA-mRNA network containing 7 miRNAs and 23 lncRNAs was established. Among them, hsa-miR-302c-5P co-targeted ADAM9, TGFA, and PDSS1 biomarkers (Fig. 6A, B).

### 3.8. The relationships between biomarkers and drugs were investigated

**Figure 7.**
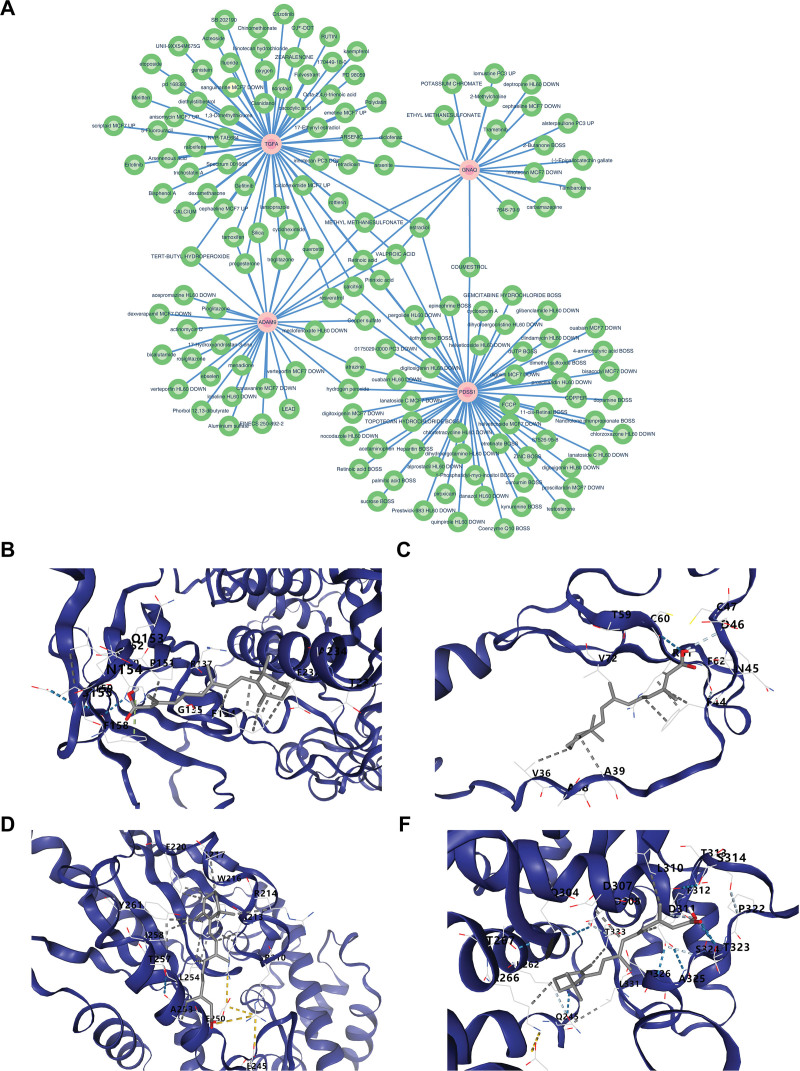
Prediction of drugs for biomarkers and validation through molecular docking. (A) Prediction of potential drugs. (B–D) Validation of molecular docking between retinoic acid and biomarkers. (B) ADAM9. (C) TGFA. (D, E) GNAQ. (F) PDSS1. GNAQ = G protein subunit alpha q, PDSS1 = decaprenyl diphosphate synthase subunit 1, TGFA = transforming growth factor alpha.

The 32 drugs corresponding to ADAM9, 64 drugs corresponding to TGFA, 21 drugs corresponding to GNAQ, and 63 drugs corresponding to PDSS1 were used for network construction (Fig. 7A and Table 1). Among them, the drug with the top-ranked scores for its related effects on the 4 biomarkers was retinoic acid (Table 2). Subsequently, the results showed that the binding energy of retinoic acid and ADAM9, TGFA, GNAQ, as well as PDSS1 was −6.4, −5.6, −6.3, and −6.3, indicating that retinoic acid can stably bind to the 4 biomarkers, which may furnish a reference for the PS management in the future (Fig. 7B–E and Table 3).

**Table 1:** Drugs information.

Term	Genes
Cycloheximide	ADAM9
Cycloheximide	TGFA
Resveratrol	ADAM9
Resveratrol	TGFA
Resveratrol	PDSS1
Retinoic acid	GNAQ
Retinoic acid	ADAM9
Retinoic acid	TGFA
Retinoic acid	PDSS1
Trametinib	GNAQ
Diclofenac	GNAQ
Diclofenac	TGFA
pd 168393	TGFA
1,3-Dimethylthiourea	TGFA
NVP-TAE684	TGFA
Phorbol 12,13-dibutyrate	ADAM9
Polydatin	TGFA
Aluminium sulfate	ADAM9
Acepromazine HL60 DOWN	ADAM9
Chinomethionate	TGFA
Troglitazone	ADAM9
Troglitazone	TGFA
Cacodylic acid	TGFA
Coenzyme Q10 BOSS	PDSS1
RUTIN	TGFA
Kynurenine BOSS	PDSS1
Acteoside	TGFA
Tamoxifen	ADAM9
Tamoxifen	TGFA
Erlotinib	TGFA
Crizotinib	TGFA
Lansoprazole	TGFA
Melitten	TGFA
Fluoride	TGFA
Silica	ADAM9
Silica	TGFA
TOPOTECAN HYDROCHLORIDE BOSS	PDSS1
Scriptaid	TGFA
FCCP	PDSS1
UNII-9XX54M675G	TGFA
170449-18-0	TGFA
Rottlerin	TGFA
Canavanine MCF7 DOWN	ADAM9
Ebselen	ADAM9
Spectrum 001666	TGFA
Alsterpaullone PC3 UP	GNAQ
Octa-2,4,6-trienoic acid	TGFA
Etretinate BOSS	PDSS1
Heparitin BOSS	PDSS1
SB 202190	TGFA
Gefitinib	TGFA
Kaempferol	TGFA
Pioglitazone	ADAM9
Arsenite	GNAQ
Arsenite	TGFA
Nandrolone phenpropionate BOSS	PDSS1
Tert-butyl hydroperoxide	TGFA
Tert-butyl hydroperoxide	ADAM9
Pirinixic acid	PDSS1
Pirinixic acid	TGFA
Lomustine PC3 UP	GNAQ
Valproic acid	GNAQ
Valproic acid	ADAM9
Valproic acid	TGFA
Valproic acid	PDSS1
Bicalutamide	ADAM9
Estradiol	TGFA
Estradiol	GNAQ
Estradiol	PDSS1
Palmitic acid BOSS	PDSS1
Dimethyl sulfoxide BOSS	PDSS1
Calcium	TGFA
O,P’-DDT	TGFA
dUTP BOSS	PDSS1
Gemcitabine hydrochloride boss	PDSS1
4-Aminobutyric acid BOSS	PDSS1
Zearalenone	TGFA
Sucrose BOSS	PDSS1
Liothyronine BOSS	PDSS1
17-Hydroxyandrostan-3-one	ADAM9
ZINC BOSS	PDSS1
Actinomycin D	ADAM9
1-Phosphatidyl-myo-inositol BOSS	PDSS1
Dihydroergocristine HL60 DOWN	PDSS1
2-Butanone BOSS	GNAQ
11-cis-Retinal BOSS	PDSS1
Dopamine BOSS	PDSS1
Cephaeline MCF7 DOWN	GNAQ
Epinephrine BOSS	PDSS1
Nocodazole HL60 DOWN	PDSS1
Coumestrol	PDSS1
Coumestrol	GNAQ
EINECS 250-892-2	ADAM9
Retinoic acid BOSS	PDSS1
Diethylstilbestrol	TGFA
Verteporfin MCF7 DOWN	ADAM9
Prestwick-983 HL60 DOWN	PDSS1
Quinpirole HL60 DOWN	PDSS1
Progesterone	TGFA
Progesterone	ADAM9
Calcitriol	PDSS1
Calcitriol	TGFA
Curcumin BOSS	PDSS1
PD 98059	TGFA
Verteporfin HL60 DOWN	ADAM9
Dihydroergotamine HL60 DOWN	PDSS1
17-Ethynyl estradiol	TGFA
Fulvestrant	TGFA
Dexverapamil MCF7 DOWN	ADAM9
Pergolide HL60 DOWN	PDSS1
Cicloheximide MCF7 UP	TGFA
Bisacodyl MCF7 DOWN	PDSS1
Sanguinarine MCF7 DOWN	TGFA
Digitoxigenin MCF7 DOWN	PDSS1
Rosiglitazone	ADAM9
Dexamethasone	TGFA
Proscillaridin MCF7 DOWN	PDSS1
Deptropine HL60 DOWN	GNAQ
Carbamazepine	GNAQ
Digoxin MCF7 DOWN	PDSS1
Hydrogen peroxide	PDSS1
Hydrogen peroxide	ADAM9
Etoposide	TGFA
Ouabain MCF7 DOWN	PDSS1
Alprostadil HL60 DOWN	PDSS1
Oxygen	TGFA
Helveticoside HL60 DOWN	PDSS1
Piroxicam	PDSS1
Ouabain HL60 DOWN	PDSS1
Helveticoside MCF7 DOWN	PDSS1
Tamibarotene	GNAQ
Atrazine	PDSS1
Atrazine	ADAM9
Lanatoside C HL60 DOWN	PDSS1
Irinotecan hydrochloride	TGFA
Proscillaridin HL60 DOWN	PDSS1
Quercetin	ADAM9
Quercetin	TGFA
Emetine MCF7 UP	TGFA
Lanatoside C MCF7 DOWN	PDSS1
Digoxigenin HL60 DOWN	PDSS1
Raloxifene	TGFA
Scriptaid MCF7 UP	TGFA
Meclofenoxate HL60 DOWN	ADAM9
Anisomycin MCF7 UP	TGFA
digitoxigenin HL60 DOWN	PDSS1
LEAD	ADAM9
Cephaeline MCF7 UP	TGFA
ARSENIC	TGFA
Cianidanol	TGFA
METHYL METHANESULFONATE	ADAM9
METHYL METHANESULFONATE	GNAQ
67526-95-8	PDSS1
Irinotecan PC3 DOWN	TGFA
Chlortetracycline HL60 DOWN	PDSS1
Menadione	ADAM9
5-Fluorouracil	TGFA
Genistein	TGFA
Danazol HL60 DOWN	PDSS1
Testosterone	PDSS1
Bisphenol A	TGFA
Irinotecan MCF7 DOWN	GNAQ
Arsenenous acid	TGFA
Chlorzoxazone HL60 DOWN	PDSS1
Glibenclamide HL60 DOWN	PDSS1
Lobeline HL60 DOWN	ADAM9
Clindamycin HL60 DOWN	PDSS1
2-Methylcholine	GNAQ
COPPER	PDSS1
POTASSIUM CHROMATE	GNAQ
Copper sulfate	ADAM9
Copper sulfate	PDSS1
(−)-Epigallocatechin gallate	GNAQ
Ethyl methanesulfonate	GNAQ
7646-79-9	GNAQ
0175029-0000 PC3 DOWN	PDSS1
Trichostatin A	TGFA
Tetradioxin	TGFA
Acetaminophen	PDSS1
Cyclosporin A	PDSS1

ADAM9 = a disintegrin and a metalloprotease9, GNAQ = G protein subunit alpha q, PDSS1 = decaprenyl diphosphate synthase subunit 1, TGFA = transforming growth factor alpha.

**Table 2:** The drug with scores for its related effects on the 4 biomarkers.

Term	Overlap	*P* value	Adjusted *P* value	Old *P* value	Old Adjusted *P* value	Odds ratio	Combined score	Genes
Cycloheximide	2/324	.00153632689226023	.0581070958765314	0	0	61.0993788819876	395.823823889515	ADAM9
Cycloheximide	2/324	.00153632689226023	.0581070958765314	0	0	61.0993788819876	395.823823889515	TGFA
Resveratrol	3/1601	.00192550589193281	.0581070958765314	0	0	34.5394242803504	215.960048730745	ADAM9
Resveratrol	3/1601	.00192550589193281	.0581070958765314	0	0	34.5394242803504	215.960048730745	TGFA
Resveratrol	3/1601	.00192550589193281	.0581070958765314	0	0	34.5394242803504	215.960048730745	PDSS1
Retinoic acid	4/4258	.00205216781636692	.0581070958765314	0	0	62968	389700.046634886	GNAQ
Retinoic acid	4/4258	.00205216781636692	.0581070958765314	0	0	62968	389700.046634886	ADAM9
Retinoic acid	4/4258	.00205216781636692	.0581070958765314	0	0	62968	389700.046634886	TGFA
Retinoic acid	4/4258	.00205216781636692	.0581070958765314	0	0	62968	389700.046634886	PDSS1
Trametinib	2025/1/12	.00239797880116383	.0581070958765314	0	0	605.606060606061	3653.69952474798	GNAQ
Diclofenac	2/427	.00265191529751145	.0581070958765314	0	0	46.0494117647059	273.186898695502	GNAQ
Diclofenac	2/427	.00265191529751145	.0581070958765314	0	0	46.0494117647059	273.186898695502	TGFA
pd 168393	2025/1/15	.00299680275873185	.0581070958765314	0	0	475.761904761905	2764.27624635263	TGFA
1,3-Dimethylthiourea	2025/1/16	.0031963509494466	.0581070958765314	0	0	444.022222222222	2551.23866220785	TGFA
NVP-TAE684	2025/1/17	.00339586923953755	.0581070958765314	0	0	416.25	2366.46263334062	TGFA
Phorbol 12,13-dibutyrate	2025/1/17	.00339586923953755	.0581070958765314	0	0	416.25	2366.46263334062	ADAM9
Polydatin	2025/1/21	.00419364337662754	.0614929701517056	0	0	332.933333333333	1822.53878648676	TGFA
Aluminium sulfate	2025/1/22	.00439301215541238	.0614929701517056	0	0	317.063492063492	1720.93824501994	ADAM9
Acepromazine HL60 DOWN	2025/1/24	.00479166001182121	.0614929701517056	0	0	289.463768115942	1545.99077797272	ADAM9
Chinomethionate	2025/1/28	.00558859694869862	.0640513455603637	0	0	246.530864197531	1278.76225296813	TGFA
Troglitazone	2/651	.00607615992677929	.0640513455603637	0	0	29.8104776579353	152.134266226074	ADAM9
Troglitazone	2/651	.00607615992677929	.0640513455603637	0	0	29.8104776579353	152.134266226074	TGFA
Cacodylic acid	1/32	.00638505560259709	.0640513455603637	0	0	214.677419354839	1084.93568598441	TGFA
Coenzyme Q10 BOSS	1/38	.00757884707428762	.0640513455603637	0	0	179.810810810811	877.907258346024	PDSS1
Rutin	1/41	.00817533951037825	.0640513455603637	0	0	166.3	799.343073344609	TGFA
Kynurenine BOSS	1/42	.008374110589681	.0640513455603637	0	0	162.235772357724	775.91049296681	PDSS1
Acteoside	1/42	.008374110589681	.0640513455603637	0	0	162.235772357724	775.91049296681	TGFA
Tamoxifen	2/802	.00913011180736516	.0640513455603637	0	0	23.995	112.684775232473	ADAM9
Tamoxifen	2/802	.00913011180736516	.0640513455603637	0	0	23.995	112.684775232473	TGFA
Erlotinib	1/46	.0091688963119721	.0640513455603637	0	0	147.785185185185	693.3989792	TGFA
Crizotinib	1/47	.00936751810595902	.0640513455603637	0	0	144.565217391304	675.192873436754	TGFA
Lansoprazole	1/48	.00956611005122314	.0640513455603637	0	0	141.482269503546	657.825862605875	TGFA
Melitten	1/53	.0105586221262761	.0657078735640596	0	0	127.846153846154	581.80387368109	TGFA
Fluoride	1/55	.0109554181035787	.0657078735640596	0	0	123.098765432099	555.658119729102	TGFA
Silica	2/898	.0113733501723967	.0657078735640596	0	0	21.3169642857143	95.4250146886896	ADAM9
Silica	2/898	.0113733501723967	.0657078735640596	0	0	21.3169642857143	95.4250146886896	TGFA
Topotecan hydrochloride BOSS	1/59	.0117486521426567	.0657078735640596	0	0	114.586206896552	509.223023481694	PDSS1
Scriptaid	1/60	.0119468861025563	.0657078735640596	0	0	112.638418079096	498.682335053748	TGFA
FCCP	1/64	.0127395238270763	.0676512644610258	0	0	105.465608465608	460.151301759579	PDSS1
UNII-9XX54M675G	1/67	.0133336891739785	.0684462710930898	0	0	100.656565656566	434.58083948016	TGFA
170449-18-0	1/70	.013927586368182	.069188654861291	0	0	96.2657004830918	411.428415366268	TGFA
Rottlerin	1/74	.0147190322987359	.0695978547118149	0	0	90.972602739726	383.778287202741	TGFA
Canavanine MCF7 DOWN	1/76	.0151145765997521	.0695978547118149	0	0	88.5377777777778	371.158834239914	ADAM9
Ebselen	1/81	.0161029164419929	.0695978547118149	0	0	82.9833333333333	342.617842286989	ADAM9
Spectrum 001666	1/86	.0170905124090214	.0695978547118149	0	0	78.0823529411765	317.735193554772	TGFA
Alsterpaullone PC3 UP	1/87	.0172879423680493	.0695978547118149	0	0	77.1705426356589	313.138460190054	GNAQ
Octa-2,4,6-trienoic acid	1/89	.0176827130771262	.0695978547118149	0	0	75.4090909090909	304.288333876803	TGFA
Etretinate BOSS	1/89	.0176827130771262	.0695978547118149	0	0	75.4090909090909	304.288333876803	PDSS1
Heparitin BOSS	1/90	.0178800538322202	.0695978547118149	0	0	74.5580524344569	300.026784668371	PDSS1
SB 202190	1/91	.0180773648602117	.0695978547118149	0	0	73.7259259259259	295.869121371115	TGFA
Gefitinib	1/95	.0188663117274591	.0708637074641147	0	0	70.5744680851064	280.207272795148	TGFA
Kaempferol	1/106	.021033464727282	.0771227040000339	0	0	63.1460317460317	243.847276836808	TGFA
Pioglitazone	1/111	.0220173467371517	.0788528231981713	0	0	60.2606060606061	229.949931986046	ADAM9
Arsenite	2/1300	.023192648733187	.0807302748411338	0	0	14.4052388289676	54.2201653154677	GNAQ
Arsenite	2/1300	.023192648733187	.0807302748411338	0	0	14.4052388289676	54.2201653154677	TGFA
Nandrolone phenpropionate BOSS	1/119	.023590015375656	.0807302748411338	0	0	56.1525423728814	210.399742949136	PDSS1
Tert-butyl hydroperoxide	2/1341	.0246091820113273	.0823872615161828	0	0	13.9335324869305	51.618661225995	TGFA
Tert-butyl hydroperoxide	2/1341	.0246091820113273	.0823872615161828	0	0	13.9335324869305	51.618661225995	ADAM9
Pirinixic acid	2/1402	.0267861703971823	.0871216474480554	0	0	13.2828571428571	48.0822101696691	PDSS1
Pirinixic acid	2/1402	.0267861703971823	.0871216474480554	0	0	13.2828571428571	48.0822101696691	TGFA
Lomustine PC3 UP	1/149	.0294706377009847	.0871216474480554	0	0	44.7027027027027	157.54845500306	GNAQ
Valproic acid	4/8312	.0298205986601112	.0871216474480554	0	0	46752	164219.013158872	GNAQ
Valproic acid	4/8312	.0298205986601112	.0871216474480554	0	0	46752	164219.013158872	ADAM9
Valproic acid	4/8312	.0298205986601112	.0871216474480554	0	0	46752	164219.013158872	TGFA
Valproic acid	4/8312	.0298205986601112	.0871216474480554	0	0	46752	164219.013158872	PDSS1
Bicalutamide	1/170	.0335712412700661	.0871216474480554	0	0	39.1065088757396	132.730834460871	ADAM9
Estradiol	3/4336	.0341175856737282	.0871216474480554	0	0	10.8444495730441	36.631925135987	TGFA
Estradiol	3/4336	.0341175856737282	.0871216474480554	0	0	10.8444495730441	36.631925135987	GNAQ
Estradiol	3/4336	.0341175856737282	.0871216474480554	0	0	10.8444495730441	36.631925135987	PDSS1
Palmitic acid BOSS	1/177	.0349352173469081	.0871216474480554	0	0	37.5378787878788	125.911800255359	PDSS1
Dimethyl sulfoxide BOSS	1/177	.0349352173469081	.0871216474480554	0	0	37.5378787878788	125.911800255359	PDSS1
Calcium	1/178	.0351299531540226	.0871216474480554	0	0	37.3239171374765	124.986644099269	TGFA
O,P′-DDT	1/178	.0351299531540226	.0871216474480554	0	0	37.3239171374765	124.986644099269	TGFA
dUTP BOSS	1/179	.0353246594928891	.0871216474480554	0	0	37.1123595505618	124.073075146765	PDSS1
Gemcitabine hydrochloride BOSS	1/179	.0353246594928891	.0871216474480554	0	0	37.1123595505618	124.073075146765	PDSS1
4-Aminobutyric acid BOSS	1/180	.0355193363615434	.0871216474480554	0	0	36.903165735568	123.170886049914	PDSS1
Zearalenone	1/184	.0362977492217357	.0871216474480554	0	0	36.0892531876138	119.671947133081	TGFA
Sucrose BOSS	1/185	.0364922787962953	.0871216474480554	0	0	35.8913043478261	118.8237111	PDSS1
Liothyronine BOSS	1/187	.0368812496049919	.0871216474480554	0	0	35.5017921146953	117.157759996032	PDSS1
17-Hydroxyandrostan-3-one	1/187	.0368812496049919	.0871216474480554	0	0	35.5017921146953	117.157759996032	ADAM9
Zinc BOSS	1/187	.0368812496049919	.0871216474480554	0	0	35.5017921146953	117.157759996032	PDSS1
Actinomycin D	1/190	.0374644849857352	.0871216474480554	0	0	34.9329805996473	114.732549200627	ADAM9
1-Phosphatidyl-myo-inositol BOSS	1/192	.0378531613980021	.0871216474480554	0	0	34.5636998254799	113.162962674115	PDSS1
Dihydroergocristine HL60 DOWN	1/192	.0378531613980021	.0871216474480554	0	0	34.5636998254799	113.162962674115	PDSS1
2-Butanone BOSS	1/196	.0386301610922472	.0871216474480554	0	0	33.8478632478632	110.131535006982	GNAQ
11-Cis-Retinal BOSS	1/197	.0388243374612762	.0871216474480554	0	0	33.6734693877551	109.395268540462	PDSS1
Dopamine BOSS	1/200	.0394066901050093	.0871216474480554	0	0	33.1608040201005	107.236060561028	PDSS1
Cephaeline MCF7 DOWN	1/201	.0396007488400252	.0871216474480554	0	0	32.9933333333333	106.532413228023	GNAQ
Epinephrine BOSS	1/207	.0407644838876256	.0884187396999203	0	0	32.0226537216829	102.470700870238	PDSS1
Nocodazole HL60 DOWN	1/212	.0417334549875237	.089263223167759	0	0	31.2559241706161	99.2829489057505	PDSS1
Coumestrol	2/1812	.0434862536875203	.0912816423168721	0	0	10.0475138121547	31.5020745294151	PDSS1
Coumestrol	2/1812	.0434862536875203	.0912816423168721	0	0	10.0475138121547	31.5020745294151	GNAQ
EINECS 250-892-2	1/223	.0438626073470684	.0912816423168721	0	0	29.6906906906907	92.8336774413382	ADAM9
Retinoic acid BOSS	1/232	.0456020005475512	.0915595192984697	0	0	28.5209235209235	88.0670129405206	PDSS1
Diethylstilbestrol	1/233	.04579511987047	.0915595192984697	0	0	28.3965517241379	87.5629749763211	TGFA
Verteporfin MCF7 DOWN	1/235	.0461812705812269	.0915595192984697	0	0	28.1509971509971	86.5694104934597	ADAM9
Prestwick-983 HL60 DOWN	1/236	.0463743019823418	.0915595192984697	0	0	28.0297872340426	86.079751561273	PDSS1
Quinpirole HL60 DOWN	1/245	.0481102664967671	.0928250314621463	0	0	26.983606557377	81.875269505007	PDSS1
Progesterone	2/1915	.0482207955647513	.0928250314621463	0	0	9.45269210663879	28.6602307507951	TGFA
Progesterone	2/1915	.0482207955647513	.0928250314621463	0	0	9.45269210663879	28.6602307507951	ADAM9
Calcitriol	2/1958	.0502583834088838	.0946934692123343	0	0	9.22290388548057	27.5818126439612	PDSS1
Calcitriol	2/1958	.0502583834088838	.0946934692123343	0	0	9.22290388548057	27.5818126439612	TGFA
Curcumin BOSS	1/257	.0504211978922819	.0946934692123343	0	0	25.703125	76.7840659489192	PDSS1
PD 98059	1/262	.0513828431042878	.0953368414224134	0	0	25.2043422733078	74.8178538658218	TGFA
Verteporfin HL60 DOWN	1/283	.0554137796166175	.101591929297132	0	0	23.3026004728132	67.4127217661119	ADAM9
Dihydroergotamine HL60 DOWN	1/293	.0573287516832011	.103866208931917	0	0	22.4931506849315	64.3068607770684	PDSS1
17-Ethynyl estradiol	1/304	.0594318564610167	.106424487151123	0	0	21.6644664466447	61.1571616109702	TGFA
Fulvestrant	1/310	.060577520270055	.107229173811362	0	0	21.2373247033441	59.5458780116698	TGFA
Dexverapamil MCF7 DOWN	1/326	.0636275072715942	.11134813772529	0	0	20.1753846153846	55.5773216056161	ADAM9
Pergolide HL60 DOWN	1/341	.0664801196025525	.115033015941495	0	0	19.2705882352941	52.2397190058011	PDSS1
Cicloheximide MCF7 UP	1/354	.0689471072185541	.117976161240637	0	0	18.5486307837583	49.6067480904856	TGFA
Bisacodyl MCF7 DOWN	1/373	.0725439052226685	.121378156829278	0	0	17.584229390681	46.1333390840342	PDSS1
Sanguinarine MCF7 DOWN	1/376	.0731108666364251	.121378156829278	0	0	17.4408888888889	45.6214981454594	TGFA
Digitoxigenin MCF7 DOWN	1/377	.0732997960072912	.121378156829278	0	0	17.3936170212766	45.4529556997994	PDSS1
Rosiglitazone	1/411	.0797062280708694	.130582543860786	0	0	15.9235772357724	40.2772165200092	ADAM9
Dexamethasone	1/421	.0815841352901515	.131476491942369	0	0	15.5365079365079	38.9363603603619	TGFA
Proscillaridin MCF7 DOWN	1/423	.0819593716004376	.131476491942369	0	0	15.4612954186414	38.6769194190327	PDSS1
Deptropine HL60 DOWN	1/435	.0842083754009061	.132621666065292	0	0	15.0245775729647	37.1777296285475	GNAQ
Carbamazepine	1/436	.084395605677913	.132621666065292	0	0	14.9892720306513	37.0570770481052	GNAQ
Digoxin MCF7 DOWN	1/449	.08682698776794	.135064203194573	0	0	14.5446428571429	35.5447478076968	PDSS1
Hydrogen peroxide	2/2673	.0890143523638516	.135805189008982	0	0	6.48633470610258	15.6901690209397	PDSS1
Hydrogen peroxide	2/2673	.0890143523638516	.135805189008982	0	0	6.48633470610258	15.6901690209397	ADAM9
Etoposide	1/461	.0890670395448521	.135805189008982	0	0	14.1565217391304	34.235649995562	TGFA
Ouabain MCF7 DOWN	1/486	.0937205754962251	.141499692415869	0	0	13.4096219931271	31.7464422995104	PDSS1
Alprostadil HL60 DOWN	1/500	.0963187548645896	.144010565525697	0	0	13.0240480961924	30.4774736823968	PDSS1
Oxygen	1/524	.100759774807303	.148924361378473	0	0	12.4110898661568	28.4836505952365	TGFA
Helveticoside HL60 DOWN	1/534	.102605360566537	.148924361378473	0	0	12.1719824890557	27.7139621316834	PDSS1
Piroxicam	1/549	.105368410753451	.148924361378473	0	0	11.8296836982968	26.6202472735742	PDSS1
Ouabain HL60 DOWN	1/549	.105368410753451	.148924361378473	0	0	11.8296836982968	26.6202472735742	PDSS1
Helveticoside MCF7 DOWN	1/555	.106471842382587	.148924361378473	0	0	11.6979542719615	26.201952044381	PDSS1
Tamibarotene	1/559	.107206896187894	.148924361378473	0	0	11.6117084826762	25.9288835265095	GNAQ
Atrazine	2/2968	.107426868025052	.148924361378473	0	0	5.74173971679029	12.80950528	PDSS1
Atrazine	2/2968	.107426868025052	.148924361378473	0	0	5.74173971679029	12.80950528	ADAM9
Lanatoside C HL60 DOWN	1/563	.107941496379256	.148924361378473	0	0	11.5266903914591	25.6603250757517	PDSS1
Irinotecan hydrochloride	1/565	.108308626457072	.148924361378473	0	0	11.48463357	25.5277044127623	TGFA
Proscillaridin HL60 DOWN	1/620	.118360372100342	.161305285871262	0	0	10.4345718901454	22.2675987498136	PDSS1
Quercetin	2/3158	.119946643491374	.162033185067295	0	0	5.33586818757921	11.3158198071829	ADAM9
Quercetin	2/3158	.119946643491374	.162033185067295	0	0	5.33586818757921	11.3158198071829	TGFA
Emetine MCF7 UP	1/637	.121450011072633	.162637406132048	0	0	10.146750524109	21.3919124877151	TGFA
Lanatoside C MCF7 DOWN	1/653	.124350481809823	.165085984471662	0	0	9.88957055214724	20.6163054563871	PDSS1
Digoxigenin HL60 DOWN	1/662	.12597883717132	.165818298499002	0	0	9.75037821482602	20.1992866401484	PDSS1
Raloxifene	1/686	.130310015837051	.169102636776936	0	0	9.3970802919708	19.1497360500547	TGFA
Scriptaid MCF7 UP	1/688	.130670219327632	.169102636776936	0	0	9.36875303250849	19.0661482383103	TGFA
Meclofenoxate HL60 DOWN	1/713	.135163328802158	.172878490200849	0	0	9.02808988764045	18.0676579879656	ADAM9
Anisomycin MCF7 UP	1/718	.136059856541621	.172878490200849	0	0	8.9628079962808	17.8777579001331	TGFA
Digitoxigenin HL60 DOWN	1/723	.136955687042231	.172878490200849	0	0	8.89843028624192	17.6909501891703	PDSS1
LEAD	1/791	.149069949258151	.186640424274433	0	0	8.10379746835443	15.4242788352171	ADAM9
Cephaeline MCF7 UP	1/811	.152608570551719	.189529998911006	0	0	7.89547325102881	14.8425343454453	TGFA
ARSENIC	1/853	.160003741264342	.19712460923767	0	0	7.48982785602504	13.725544563753	TGFA
Cianidanol	1/870	.162983221685965	.199201715393957	0	0	7.33678557729191	13.3097215414915	TGFA
Methyl methanesulfonate	2/3864	.170429440978546	.206662471737764	0	0	4.17762817193164	7.39203692494949	ADAM9
Methyl methanesulfonate	2/3864	.170429440978546	.206662471737764	0	0	4.17762817193164	7.39203692494949	GNAQ
67526-95-8	1/953	.177416438588813	.213454152677165	0	0	6.66806722689076	11.530792256051	PDSS1
Irinotecan PC3 DOWN	1/999	.185334725485974	.221252307944496	0	0	6.34535738142953	10.6956821278963	TGFA
Chlortetracycline HL60 DOWN	1/1050	.194046721066909	.229870731110031	0	0	6.02065459167461	9.87180434378061	PDSS1
Menadione	1/1140	.209250153357172	.245988729900798	0	0	5.51858355282411	8.63230545685913	ADAM9
5-Fluorouracil	1/1202	.219597782508191	.256197412926223	0	0	5.21648626144879	7.90797234422962	TGFA
Genistein	1/1231	.224402786960448	.258275637149566	0	0	5.08563685636856	7.59953166984805	TGFA
Danazol HL60 DOWN	1/1233	.224733346610661	.258275637149566	0	0	5.07683982683983	7.57891315018041	PDSS1
Testosterone	1/1247	.227044306710741	.258647700375281	0	0	5.01605136436597	7.43684839887061	PDSS1
Bisphenol A	1/1262	.229514598119547	.258647700375281	0	0	4.95241871530531	7.28891362126245	TGFA
Irinotecan MCF7 DOWN	1/1272	.231158166312747	.258647700375281	0	0	4.91083136637818	7.19266438488164	GNAQ
Arsenenous acid	1/1283	.232963052766599	.258647700375281	0	0	4.86583463338534	7.08891482556854	TGFA
Chlorzoxazone HL60 DOWN	1/1286	.23345474254652	.258647700375281	0	0	4.85369649805447	7.06099770078215	PDSS1
Glibenclamide HL60 DOWN	1/1333	.241127063674463	.26523977004191	0	0	4.67067067067067	6.64370791699849	PDSS1
Lobeline HL60 DOWN	1/1510	.26950494133371	.294352914648166	0	0	4.08371990280539	5.35444511831442	ADAM9
Clindamycin HL60 DOWN	1/1626	.287666106092366	.31197591787482	0	0	3.76841025641026	4.69526894254378	PDSS1
2-Methylcholine	1/1666	.293849348420552	.316453144452903	0	0	3.66986986986987	4.4944458235449	GNAQ
Copper	1/1688	.297232924749405	.317874100079225	0	0	3.61766449318317	4.38909233626793	PDSS1
Potassium chromate	1/1897	.32877383893695	.349180491008898	0	0	3.18213783403657	3.53976298079984	GNAQ
Copper sulfate	2/6016	.349706606160354	.36886861197736	0	0	2.32490854672431	2.44269014379401	ADAM9
Copper sulfate	2/6016	.349706606160354	.36886861197736	0	0	2.32490854672431	2.44269014379401	PDSS1
(−)-Epigallocatechin gallate	1/2114	.360386156722584	.377547402280803	0	0	2.82110743019404	2.87916346507452	GNAQ
Ethyl methanesulfonate	1/2315	.388658926322738	.404415369281768	0	0	2.54710458081245	2.40714912102678	GNAQ
7646-79-9	1/3094	.489471370587577	.505896584365684	0	0	1.82164026295937	1.30143318843779	GNAQ
0175029-0000 PC3 DOWN	1/3326	.51692593034973	.530710621825723	0	0	1.67127819548872	1.10280241503337	PDSS1
Trichostatin A	1/3584	.546140424972012	.556990896991323	0	0	1.52693273792911	0.923609773130768	TGFA
Tetradioxin	1/3768	.566151099498488	.573600456070837	0	0	1.43606760463676	0.816970640459268	TGFA
Acetaminophen	1/4135	.604080369201773	.608028606908974	0	0	1.27898726011934	0.644671006692784	PDSS1
Cyclosporin A	1/4825	.668598676795603	.668598676795603	0	0	1.04836926478718	0.42204336110007	PDSS1

*P* value: Nominal *P* value from the enrichment test, indicating the probability that the observed association between the drug and biomarkers occurred by chance (without multiple testing correction); Adjusted *P* value: *P* value corrected for multiple hypothesis testing, controlling the false discovery rate across all comparisons; Old *P* value: Original uncorrected *P* value from a prior analysis or preliminary dataset; Old Adjusted *P* value: Previously adjusted *P*-value; Odds ratio: Measure of association strength, representing the odds of biomarker genes being targeted by the drug compared to random expectation (values > 1 suggest enrichment); Combined score: Integrated metric combining *P*-value, fold enrichment, and/or other statistical features to rank terms.

ADAM9 = a disintegrin and a metalloprotease9, GNAQ = G protein subunit alpha q, PDSS1 = decaprenyl diphosphate synthase subunit 1, TGFA = transforming growth factor alpha.

**Table 3:** Binding information.

ID	Cavities_volume	Center_x	Center_y	Center_z	Size_x	Size_y	Size_z	Score
ADAM9	811	13.758	14.879	−10.485	25	25	25	−6.4
TGFA	71	−14.198	−5.633	30.552	25	25	25	−5.6
GNAQ	107	10.553	11.885	140194	25	25	25	−6.3
PDSS1	222	−12.825	−6.823	19.383	25	25	25	−6.3

Cavities_volume: Volume (Å³) of the predicted binding cavity or pocket on the protein surface; center_x, center_y, center_z: Spatial coordinates (Å) of the cavity’s geometric center within the 3D protein structure; size_x, size_y, size_z: Dimensions (Å) of the bounding box enclosing the cavity, representing its spatial extent along each axis; score: Binding affinity score (docking score or energy value in kcal/mol); negative values indicate stronger predicted binding.

ADAM9 = a disintegrin and a metalloprotease9, GNAQ = G protein subunit alpha q, PDSS1 = decaprenyl diphosphate synthase subunit 1, TGFA = transforming growth factor alpha.

### 3.9. Validation of biomarkers expression in clinical samples

**Figure 8.**
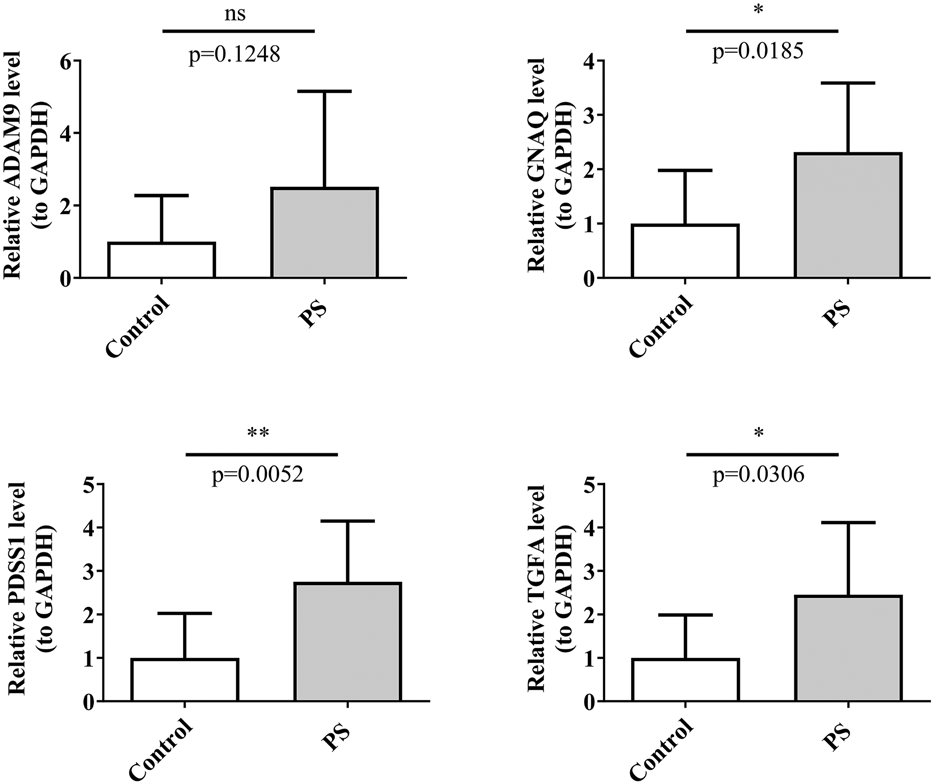
Validation results of 4 biomarkers qRT-PCR. qRT-PCR = quantitative real-time polymerase chain reaction.

To verify the reliability of the bioinformatics analysis results, qRT-PCR was undertaken through clinical samples to further analyze the expression of 4 biomarkers. The results showed that the expression of TGFA (*P* = .0306), GNAQ (*P* = .0185) and PDSS1 (*P* = .0052) were significantly higher in the PS samples and were consistent with the results of the bioinformatics analysis, whereas there was no significant difference in the expression of ADAM9 between the control and PS samples. In conclusion, TGFA, GNAQ, and PDSS1 might serve as potential therapeutic targets for PS (Fig. 8A–D).

## 4. Discussion

Children have a greater risk of developing sepsis after infection than any other age group, and their mortality rate remains high despite improved management of sepsis. Genes related to glycolysis and MP play a crucial role in cell growth, death, and metabolic regulation during tumorigenesis and progression. However, the mechanism of this simultaneous targeting of genes in both directions in the occurrence and development of PS is not clear. In this study, we identified biomarkers (ADAM9, TGFA, GNAQ, and PDSS1) associated with glycolysis and MP in PS, including the biological functions, pathways, immune microenvironment and their molecular regulation mechanism, and the expression of biomarkers in blood samples was confirmed by qRT-PCR. It is expected that this study will provide a new theoretical basis for the clinical diagnosis and treatment of PS, estimate the condition with the help of relevant genetic test results, and guide the more active and effective diagnosis and treatment.

In this study, we found that the candidate signature genes were associated with peroxisomes and MAPK signaling pathways.We identified 603 DEGs through differential expression analysis (*P* < .05, |*log*_2_ (FC)| > 1), including 513 upregulated genes and 90 downregulated genes. Additionally, WGCNA analysis (|*r*| > 0.3 and *P* < .05) identified 594 key module genes associated with glycolysis and MP. Further PPI network analysis of the 43 candidate characteristic genes in the intersection of these 2 gene sets revealed an average node degree of 0.81 (*P* = .028), indicating significant interactions between proteins encoded by these candidate genes. Through GO and Kyoto Encyclopedia of Genes and Genomes functional enrichment analysis (*P* < .05), we found that the candidate characteristic genes were significantly associated with peroxisome and MAPK signaling pathways. Peroxisomes are subcellular organelles that play a central role in human physiology by catalyzing a range of unique metabolic functions.^[24]^ It has been shown that peroxisome dysfunction plays an important role in viral infections, brain diseases, and sepsis.^[25]^ PPARα is the peroxisome proliferator activated receptor-α, studies have demonstrated that Gypenoside XLIX can alleviate sepsis-associated encephalopathy by targeting PPARα, and experimental studies by Standage et al have shown that PPARα levels in leukocytes are reduced in pediatric patients with sepsis, and similar results have been observed in an animal model in which PPARα deficiency leads to fatty acid oxidation in the septic heart.^[25,26]^ In addition, when peroxisomal dysfunction occurs it leads to Zellweger syndrome, which is most severe and life-threatening in neonatal sepsis.^[27]^

In sepsis, the expression and phosphorylation of 6-phosphofructo-2-kinase/fructose-2,6-bisphosphatase 3, a key enzyme in postinfectious glycolysis, can be attenuated by inhibition of p38 in the MAPK signaling pathway to reduce the development of sepsis.^[28]^

Four biomarkers, ADAM9, TGFA, GNAQ, and PDSS1, were obtained. Through cross-validation using LASSO regression (λ = 0.003947842) and SVM-RFE machine learning algorithms, we successfully identified 4 key biomarkers (ADAM9, TGFA, GNAQ, and PDSS1). Expression validation in 2 independent datasets (GSE26440 and GSE13904) revealed significant differences (*P* < .05), with the upregulated expressions of TGFA (*P* = .0306), GNAQ (*P* = .0185), and PDSS1 (*P* = .0052) consistent with qRT-PCR verification results. The linear model constructed using these biomarkers demonstrated excellent predictive performance (AUC = 0.965, HL test *P* = .671), indicating their potential significant value in sepsis diagnosis and treatment. A disintegrin and a metalloprotease9 (ADAM9) is a membrane-anchored protein that participates in a variety of physiological functions, and influences the developmental process, inflammation, and degenerative diseases.^[29]^ In recent years, the crucial roles of ADAM9 in tumor proliferation, angiogenesis, metastasis, and immune evasion have been broadly studied.^[29]^ In inflammation, ADAM9 contributes to monocyte fusion, mediating the conversion of monocytes-macrophages to multinucleated giant cells as a response to foreign bodies or bacteria; the resulting granulomatous lesions help to isolate the pathogens and also enhance phagocytotic activity. ADAM9 also stimulates the inflammatory process through polymorphonuclear leukocytes, macrophages, and epithelial cells in some inflammatory diseases, such as acute lung injury or chronic obstructive pulmonary disease.^[29]^ Notably, toll-like receptors (TLRs), as key recognition receptors in the innate immune system, may play a significant role in immune regulation of pediatric sepsis. TLRs can recognize pathogen-associated molecular patterns and damage-associated molecular patterns, activating transcription factors like NF-κB through MyD88-dependent or nondependent pathways to induce pro-inflammatory cytokines such as interleukins (ILs).^[30]^ These pro-inflammatory cytokines recruit additional immune cells (e.g., neutrophils, monocytes, etc) to infection sites, creating a local inflammatory environment that restricts pathogen spread and initiates adaptive immune responses.^[31]^ Furthermore, different types of TLRs exhibit distinct recognition patterns: surface-bound TLR4 recognizes gram-negative bacterial lipopolysaccharides, while endosomal TLRs 3/7/8/9 target viral nucleic acids. This differential recognition pattern provides potential insights for studying the heterogeneous immune responses in pediatric sepsis.^[32]^ Similar to the central role of TLRs in immune recognition and inflammatory regulation, ADAM9, as a crucial extracellular matrix metalloproteinase, participates in inflammatory responses by regulating monocyte-macrophage transformation.^[29]^ These 2 proteins exhibit functional overlap and complementarity in critical aspects such as initiating inflammatory responses, recruiting immune cells, and recognizing and responding to pathogens. This suggests that ADAM9 may exert synergistic effects with TLR-mediated immune activation, collectively regulating the immune process in pediatric sepsis.

Unfortunately, there are few studies on ADAM 9 in PS, but, based on its effects in inflammation, we hypothesize that ADAM 9 also plays a critical role in PS,targeting ADAM9 might provide a new avenue for alleviating the inflammatory response in PS.

Transforming growth factor alpha (TGFA) is a polypeptide growth factor with a high affinity for the EGFR.^[33]^ The EGFR family is a subclass of receptor tyrosine kinase proteins.^[34]^ Seven known ligands bind and activate EGFRs: EGF, TGFA, and others.^[35]^ After binding to EGFR, the intrinsic tyrosine kinase activity is activated, thus initiating a series of signaling pathways such as Ras-MAPK, PI3K-AKT, etc, promoting cell growth, proliferation and survival.^[36]^ Based on this relationship, we hypothesized that TGFA could regulate the MAPK signaling pathway through EGFR, ranging from regulating PS.

Mutations in G protein subunit alpha q (GNAQ) are commonly found in tumors (e.g., uveal melanoma) and are considered valuable potential therapeutic targets.^[37]^ PDSS1 is often associated with immune infiltration; in hepatocellular carcinoma, patients with higher PDSS1 expression have a shorter overall survival, and it is also a new target for immunotherapy.^[38]^ In addition, PDSS1 plays an important role in inflammatory diseases.^[38–40]^ Similarly, an inflammatory response exists in PS, and it is hypothesized that targeting PDSS1 has a regulatory effect on the inflammatory response in PS.^[41]^ However, unfortunately, there is no report on GNAQ and PDSS1 in PS, and we found a relationship between the 2 for the first time, and more experiments will be needed will be needed to explore the mechanism of action of the 2 in PS.

In order to further explore the mechanism of biomarkers in biological pathways, we found through GSEA enrichment analysis that 4 biomarkers were significantly enriched in Fcγ receptor-mediated phagocytosis pathway (FcγR, *P* < .05), indicating that FcγR-mediated phagocytosis may play an important role in the regulation of inflammatory response in sepsis. FcγR is associated with inflammation and the release of toxic mediators from cells; in response to inflammatory stimuli, immune complexes recruit macrophages via Fcγ receptors, which mediate tissue damage as well as phagocytosis and/or the release of inflammatory or cytotoxic mediators.^[42]^ Taken together, insight into the mechanisms regulating these biological pathways and pathways in the development of PS, through the activation or inactivation of regulatory pathways may provide potential biological pathways for the treatment of PS. The biomarker functional interaction analysis conducted through GeneMANIA further expanded its protein interaction network, revealing multiple interaction relationships between these biomarkers and 20 function-related genes (including 77.64% physical interactions and 8.01% co-expression) with significant correlations (*P* < .05). Pathway enrichment analysis demonstrated that these interacting genes were significantly involved in the ERBB signaling pathway, EGFR signaling pathway, and its regulatory processes (FDR < 0.05). Previous studies have shown that upregulation of TGFA can activate the EGFR signaling pathway to inhibit LCN2 expression, ultimately promoting epithelial cell proliferation.^[43]^ Similarly, in sepsis, TGFA may disrupt immune balance by overactivating the EGFR signaling pathway, leading to abnormal cell proliferation responses that influence disease progression. Pediatric sepsis is a complicated condition characterized by life-threatening organ failure resulting from a dysregulated host response to infection in children.^[44]^ In this study, through Wilcoxon rank sum test analysis, we found that there were significant differences in the infiltration level of 22 kinds of immune cells between PS group and normal group (*P* < .05). Further correlation analysis revealed that 4 biomarkers (ADAM9, TGFA, GNAQ, and PDSS1) showed significant positive correlations with activated dendritic cells (Spearman *r* > 0.3, *P* < .05), while demonstrating a marked negative correlation with activated B cells (Spearman *r* < −0.3, *P* < .05). These findings suggest that these biomarkers may contribute to the immune pathophysiology of sepsis by modulating the activation status of specific immune cell subsets, such as dendritic cells and B cells. Dysregulation of immunity is an important feature of PS.^[44]^ In this study, by comparing the differences in the infiltration of immune cells in PS and normal samples, differences were found in 22 cells, such as, macrophage and monocyte. Notably, the biomarkers showed a significant positive correlation with activated dendritic cells and a significant negative correlation with activated B cells. Monocyte is the first line of defence against microorganisms. Monocyte distribution width has emerged as a promising biomarker of sepsis, especially in acute settings7 such as emergency departments and intensive care units.^[45]^ Macrophage phagocytosis of dying cells is essential for tissue regression and repair after injury or inflammation.^[46]^ Similar to macrophages, dendritic cells are also the body’s innate immune cells, and both switch to metabolic reprogramming when stimulated by pro-inflammatory factors.^[47]^ One study found that ADAM9 in lung cancer cells releases inflammatory factors and regulates cholesterol metabolism, leading to dysfunction of activated dendritic cells.^[48]^ Beyond innate immune cells, the complex network composed of soluble toll-like receptors (sTLRs) and the interleukin family (ILs) may play a crucial role in regulating immune balance during sepsis. As essential immunoglobulins, ILs are not only secreted by white blood cells but also coordinate with TLR signaling through their TIR domain, jointly participating in maintaining homeostasis and controlling tumor and infectious disease progression.^[49]^ The significant positive correlation between biomarkers and activated dendritic cells suggests that the TLR/IL axis may deeply engage in sepsis’s immune regulation. Studies have demonstrated that TLRs serve as the body’s first line of defense against pathogens, recognizing various molecules expressed by pathogens and released by damaged/dead cells. Widely distributed across epithelial cells to immune cells, they act as a central hub bridging innate and adaptive immunity. When activated, TLRs mediate the expression of adhesion proteins and downstream kinases, thereby inducing key pro-inflammatory mediators.^[50]^ In sepsis – a severe systemic infection – immune system balance and coordination are critical for disease resolution. Genes such as ADAM9 may regulate the TLR signaling pathway through a variety of mechanisms, such as affecting the level of soluble TLR, which may indirectly affect the functional status of dendritic cells, including their antigen presentation ability and cytokine secretion pattern,^[51]^ and ultimately affect the occurrence and progression of sepsis at multiple levels.In addition, some studies have focused on the role of B cells in sepsis, as one of the important cells of innate immunity, B cells in sepsis in an important role.^[52]^ Targeting biomarkers to modulate the degree of infiltration of these immune cells in PS may be able to balance the excessive inflammatory response in PS as well as late immunosuppression. Moreover, targeting biomarkers to modulate the degree of infiltration of these immune cells in PS may be able to balance the excessive inflammatory response in PS with advanced immune suppression.

Recently, the role of the lncRNA-miRNA-mRNA regulatory network has been extensively studied in various diseases, and lncRNAs, among others, have been used as biomarkers to predict diseases.^[53–56]^ We also established a lncRNA-miRNA-mRNA regulatory network based on biomarkers, in which, Hsa-miR-30a-5p coregulates ADAM 9, GNAQ, and PDSS1. It has been found that miR-30a-5p can inhibit the progression of lung squamous cell carcinoma through the autophagy pathway.^[57]^ In addition, miR-30a-5p is differentially expressed in inflammatory diseases.^[58]^ This study is the first to identify an important role of Hsa-miR-30a-5p in PS, and if it is used to modulate the expression of multiple biomarkers to regulate glycolysis and MP in PS, it will be a multiplicative approach to the treatment and diagnosis of PS.

This study identified 4 key biomarkers, ADAM9, TGFA, GNAQ, and PDSS1, through comprehensive bioinformatics analysis and experimental validation. Functional analysis revealed that these molecules play significant roles in glycolysis, MP, and inflammatory responses. Specifically, ADAM9, as a membrane-bound metalloproteinase, not only participates in inflammatory responses by promoting monocyte-macrophage fusion (*P* < .05), but its expression level also shows a significant positive correlation with activated dendritic cell infiltration (*r* = 0.35, *P* = .008), suggesting its potential regulatory role in immune cell activation during sepsis. TGFA may activate downstream pathways such as Ras-MAPK and PI3K-AKT through the EGFR signaling pathway, a potential metabolic role in sepsis consistent with its significant enrichment in MAPK signaling pathways identified by GSEA analysis (*P* < .05). As a G protein α subunit, GNAQ mutations are associated with tumor development. Immunoinfiltration analysis showed its high expression was significantly correlated with increased MDSC cell infiltration (*r* = 0.42, *P* = .002), indicating its potential involvement in sepsis pathogenesis through regulation of myeloid immune cell function. PDSS1, closely related to lysosomal function, exhibits negative correlation with B cell activation (*r* = −0.38, *P* = .005). Four biomarkers were significantly enriched in the FcγR-mediated phagocytosis pathway. qRT-PCR validation demonstrated significantly elevated expression of TGFA (*P* = .0306), GNAQ (*P* = .0185), and PDSS1 (*P* = .0052) in septicemia patients. These statistical findings suggest that these biomarkers may play potential roles in pediatric sepsis research, indicating significant clinical value. The study will become the prevention and treatment of PS occurrence and development, the prognosis of the new target, is the new direction of PS prevention and control, provide scientific evidence for the treatment of PS, and give more positive and effective diagnosis and treatment of disease. This study demonstrates the following innovations and advantages: First, it employs an integrated multi-omics approach combining differential expression analysis, WGCNA network construction, and machine learning algorithms to systematically identify biomarkers associated with glycolysis and MP in pediatric sepsis. The findings were validated across 2 independent datasets and further confirmed through qRT-PCR experiments, demonstrating rigorous design and reliable results. Additionally, the study not only systematically analyzed gene expression profiles but also explored immune microenvironment changes and regulatory networks, providing multidimensional insights into sepsis pathogenesis. However, limitations include: bioinformatics analysis primarily based on publicly available transcriptome data, with experimental validation required for specific molecular mechanisms of biomarkers; limited clinical sample size (n = 20) in qRT-PCR verification necessitating expanded studies for broader applicability; and while significant correlations between biomarkers and immune cell infiltration were identified, causal relationships and regulatory mechanisms require further experimental investigation.

## 5. Conclusions

This study identified 4 biomarkers, ADAM 9, TGFA, GNAQ and PDSS1, which provide potential new targets for the prevention, effective diagnosis and treatment of PS.

## Acknowledgments

We would like to express our sincere gratitude to all individuals and organizations who supported and assisted us throughout this research. Special thanks to the following authors: Hang Yu, Hui Sun, Jing Huang, Xiaoping Zhu. In conclusion, we extend our thanks to everyone who has supported and assisted us along the way. Without your support, this research would not have been possible.

## Author contributions

**Conceptualization:** Hang Yu, Xiaoping Zhu.

**Data curation:** Hang Yu, Hui Sun.

**Project administration:** Xiaoping Zhu.

**Supervision:** Xiaoping Zhu.

**Validation:** Hang Yu, Hui Sun, Jing Huang.

**Visualization:** Hang Yu, Hui Sun.

**Writing – original draft:** Hang Yu.

**Writing – review & editing:** Hang Yu, Hui Sun, Jing Huang, Xiaoping Zhu.

## Supplementary Material




