# Supplementary material for: Exploring the biomarkers for diagnostic accuracy associated with glycolysis and macrophage polarization in pediatric sepsis and performing mechanistic studies
Source: Medicine (Baltimore). 2025 Nov 21;104(47):e46074. doi: 10.1097/MD.0000000000046074 (PMC12643628; doi:10.1097/MD.0000000000046074)
Supplement: Supplementary file 1 [file medi-104-e46074-s001.pdf]

Ethics approval and consent to participate

I certify that the research study titled “Exploring the biomarkers associated with glycolysis and macrophage polarization in pediatric sepsis and performing mechanistic studies” has been approved by the relevant ethics committee.

Ethics Review Approval

Affiliated Hospital of Guizhou Medical University， We hereby approve the submission of the article entitled "Exploring the biomarkers associated with glycolysis and macrophage polarization in pediatric sepsis and performing mechanistic studies" by Hang Yu at Department of pediatrics, The Affiliated Hospital of Guizhou Medical University, Guiyang, Guizhou, China. as its content complies with the relevant regulatory requirements on medical ethics and does not infringe on patient privacy or involve any commercial interest.

Ethics Committee, Affiliated Hospital of Guizhou Medical University

2024-07-29
