# Supplementary material for: Exploring the biomarkers for diagnostic accuracy associated with glycolysis and macrophage polarization in pediatric sepsis and performing mechanistic studies
Source: Medicine (Baltimore). 2025 Nov 21;104(47):e46074. doi: 10.1097/MD.0000000000046074 (PMC12643628; doi:10.1097/MD.0000000000046074)
Supplement: Supplementary file 2 [file medi-104-e46074-s002.pdf]

Graphical Abstract:

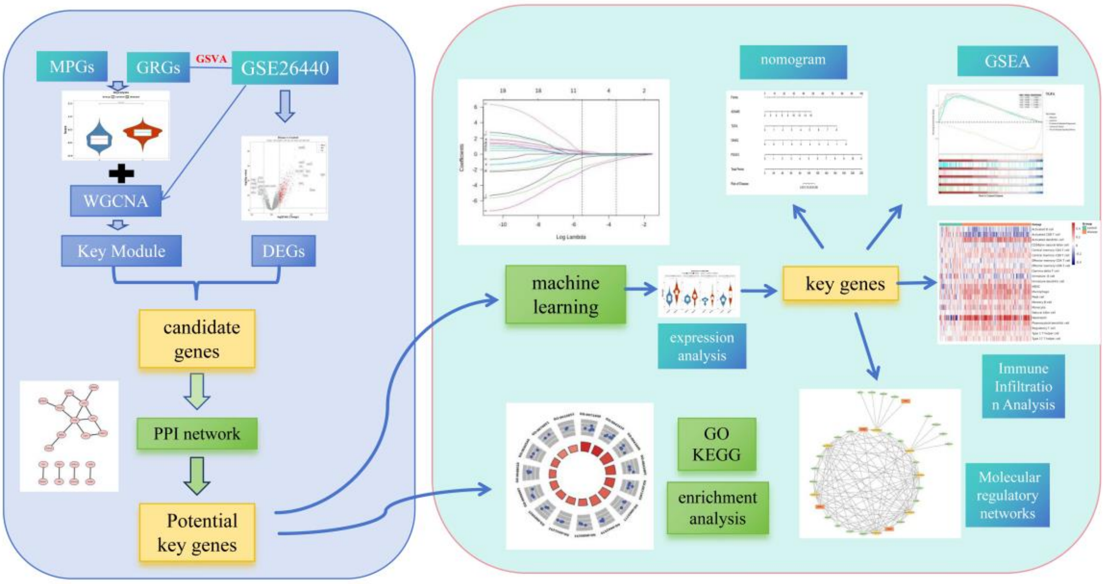

Supplementary Figure S1 Research methodology workflow.

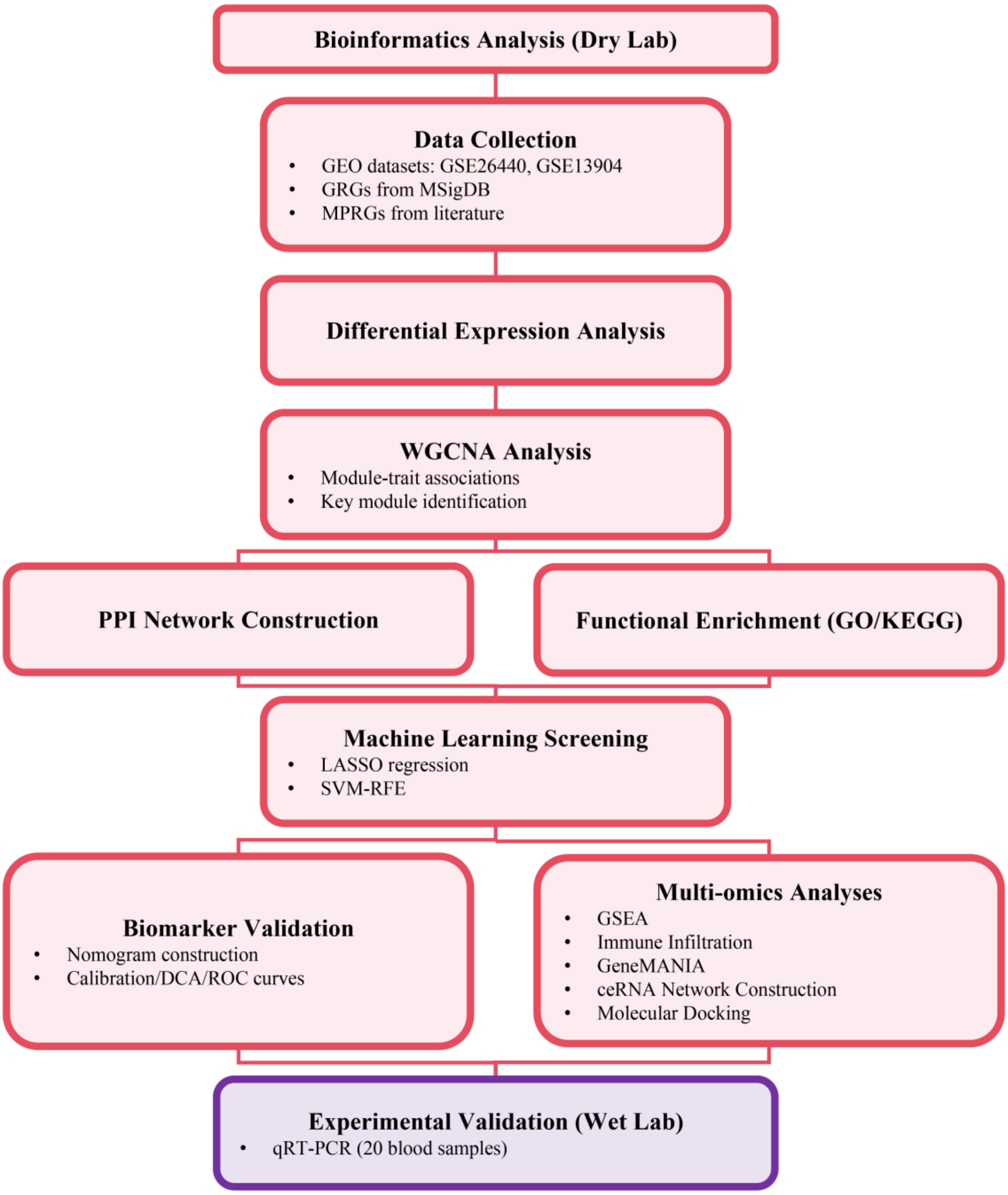

Supplementary Figure S2 Research findings workflow.

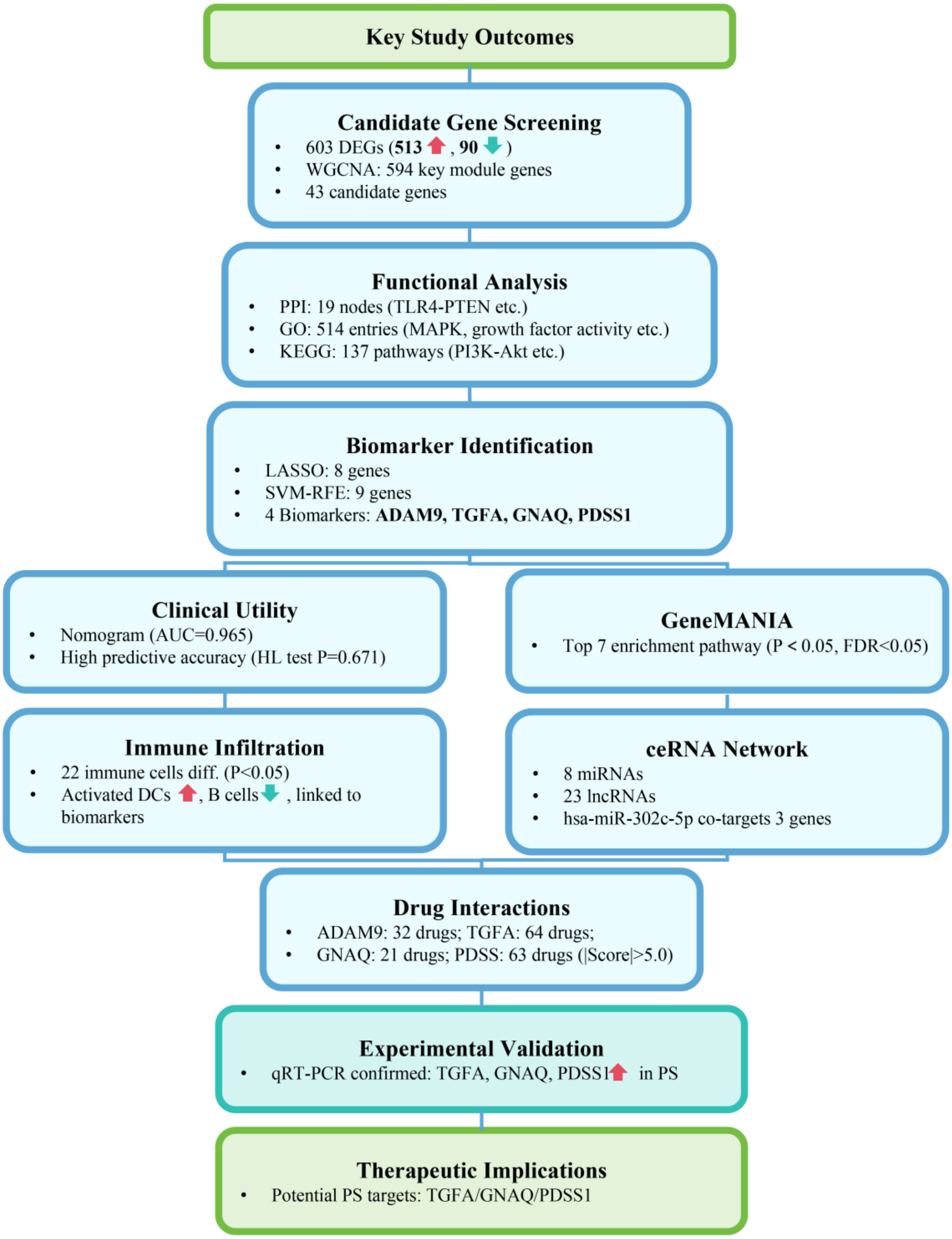

Supplementary Table 1 PCR Sequences

Primers	Sequences
ADAM9 F	CGCTCGGAGTGAACCTGT
ADAM9 R	TCGAACAGGACACGGAGGAA
TGFA F	GGTTCCCTGCGAAGACTTGA
TGFA R	GGGAGGCGTATATGTGAGGC
GNAQ F	CAGTGTCCAAACCGGCAATG
GNAQ R	ACCAACCAATGTTCCTGGCA
PDSS1 F	CGGTTTCATCACACAACCCC
PDSS1 R	AAACTGGACATCGGATCGGG
internal reference-GAPDH F	CGAAGGTGGAGTCAACGGATTT
internal reference-GAPDH R	ATGGGTGGAATCATATTGGAAC

Note:
F: Forward primer; R: Reverse primer
